# Forecasting local hospital bed demand for COVID-19 using on-request simulations

**DOI:** 10.1038/s41598-023-48601-8

**Published:** 2023-12-03

**Authors:** Raisa Kociurzynski, Angelo D’Ambrosio, Alexis Papathanassopoulos, Fabian Bürkin, Stephan Hertweck, Vanessa M. Eichel, Alexandra Heininger, Jan Liese, Nico T. Mutters, Silke Peter, Nina Wismath, Sophia Wolf, Hajo Grundmann, Tjibbe Donker

**Affiliations:** 1 Institute for Infection Prevention and Hospital Hygiene, Freiburg University Hospital, Freiburg Im Breisgau, Germany; 2 Section for Hospital Hygiene and Environmental Health, Center for Infectious Diseases, Heidelberg University Hospital, Heidelberg, Germany; 3 Unit of Hospital Hygiene, Mannheim University Hospital, Mannheim, Germany; 4 Institute of Medical Microbiology and Hygiene, Tübingen University Hospital, Tübingen, Germany; 5 Institute for Hygiene and Public Health, Medical Faculty University of Bonn, Bonn, Germany

**Keywords:** Computer modelling, Population dynamics, Stochastic modelling, Time series, Computational models, Computational platforms and environments, Statistical methods, Vaccines, Infectious diseases, Viral infection, Epidemiology

## Abstract

Accurate forecasting of hospital bed demand is crucial during infectious disease epidemics to avoid overwhelming healthcare facilities. To address this, we developed an intuitive online tool for individual hospitals to forecast COVID-19 bed demand. The tool utilizes local data, including incidence, vaccination, and bed occupancy data, at customizable geographical resolutions. Users can specify their hospital’s catchment area and adjust the initial number of COVID-19 occupied beds. We assessed the model’s performance by forecasting ICU bed occupancy for several university hospitals and regions in Germany. The model achieves optimal results when the selected catchment area aligns with the hospital’s local catchment. While expanding the catchment area reduces accuracy, it improves precision. However, forecasting performance diminishes during epidemic turning points. Incorporating variants of concern slightly decreases precision around turning points but does not significantly impact overall bed occupancy results. Our study highlights the significance of using local data for epidemic forecasts. Forecasts based on the hospital’s specific catchment area outperform those relying on national or state-level data, striking a better balance between accuracy and precision. These hospital-specific bed demand forecasts offer valuable insights for hospital planning, such as adjusting elective surgeries to create additional bed capacity promptly.

## Introduction

The speed of community transmission of virulent pathogens during epidemics or pandemics has the potential to overwhelm healthcare systems^1^. Hospital beds are usually in limited supply, because most hospitals run at near-full capacity under normal circumstances. This sudden increase in admissions, in combination with a potential long length of stay of patients in hospital, can put sudden pressure on the limited number of beds available^2^.

Forecasting of bed demand is therefore essential to hospitals in order to prevent them from becoming overwhelmed by incoming patients. The capacity of hospitals to admit COVID-19 patients is not static, and available beds, as well as nursing staff, doctors, and personal protective equipment can, to a certain degree, be steered. For instance, cancellation of elective surgeries can free up resources needed to cater for an increasing number of admitted COVID-19 patients. However, this can’t be done overnight, and a preparation period needs to be considered. Forecasting models help bridge the gap between the current situation and the situation after the preparation period, thus forming a critical part of the decision process.

Several online dashboards which provide critical information such as the number of cases, hospitalizations, etc. have been established to facilitate access to COVID-19 related data^3,4^. One prominent example is the Johns Hopkins University dashboard which mostly focuses on the visualisation of data at the country, region, or city level^5^. Some online accessible dashboards additionally offer forecasts of the hospital bed demand^6,7^. However, many forecasting models focus on large geographical areas, such as entire countries, states, or provinces, for their predictions^6^. This creates a problem for the hospitals trying to interpret what these predictions entail for their own hospital planning, as the proportion of the nationally required beds that should be freed up locally is largely unknown. Furthermore, local epidemic dynamics may deviate substantially from the national trend^8^, causing the national level forecast to over- or underestimate the local epidemic growth, and consequently the local future bed demand.

Relying only on national-level models to produce forecasts of the total bed demand may result in an excessive transfer of patients when local bed demand does not align with the local bed supply. This can happen when certain localities experience sudden increases in incidence that were absent elsewhere, for instance after super-spreading events. Such patient transfers have happened on a regular basis during the COVID-19 pandemic, sometimes over large distances and across country borders^9,10^. Some of these transfers could be avoided if the local bed supply is updated reacting to the local trends and forecasts.

However, local forecasting comes with a unique set of challenges. Because numbers are lower, actual bed occupancy in a single hospital is strongly influenced by stochastic events. The discharge of a couple of patients from an Intensive Care Unit (ICU) matters a lot for the bed supply of a single hospital. Uncertainty about future numbers of beds is therefore greater when looking at a single hospital compared to an entire country. To be widely applicable, the model needs to be able to produce bespoke forecasts for each hospital in a country for which basic levels of data are available. This capability requires a flexible forecasting system because of the vast number of potential combinations of parameter choices for all individual hospitals, in particular concerning the possible choices for catchment areas per hospital, the number of currently occupied beds, and patients’ lengths of stay on various wards. Such a variety of parameters enables the extreme forecast flexibility required to produce ad-hoc reports and forecasts for all situations of specific hospitals.

We made the use of this model accessible for non-technical users by creating an online interactive dashboard. To our knowledge, this is the first online accessible tool for predicting the bed demand on a regional level for Germany. Our platform allows individual users (i.e., hospital managers) to enter the data and parameters related to their specific context and requirements and produce ad-hoc bed occupancy estimates and predictions. End user-specific forecasting of bed demand is a unique concept, as on-request forecasting is often avoided because of the computational challenges of multiple concurrent user modelling platforms.

Here we show how some of these computational challenges can be solved. Careful streamlining of the model and its technical implementation can help produce epidemic forecasts in a reasonable time, allowing users to explore the potential future epidemic trajectory without being restricted to the developer’s viewpoint.

We will first discuss the forecast model itself, its data and parameter requirements, as well as the different modules it contains. Each module is designed to guide the user through the steps and assumptions needed to produce the forecast. Then we will discuss the technical implementation of the online dashboard and the technologies that ensure usability for multiple concurrent users. Finally, we investigate the forecasting performance of this tool for several hospitals during the COVID-19 pandemic in Germany. In particular, we focus on the influence of the choice to use local, regional, or national COVID-19 incidence data on the precision and accuracy of the forecast.

The code used to implement the dashboard, in the version used to prepare this manuscript, is available at: https://github.com/QUPI-IUK/Bed-demand-forecast/releases/tag/v.0.5.6.

## Methods

### General model structure

**Figure 1:**
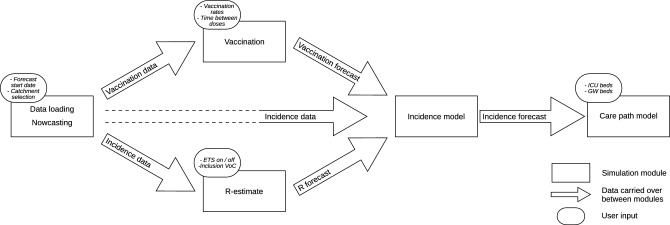
Model structure showing each of the modules, the data carried over between modules, and the possible user input in each module.

The complete forecast model consists of 5 main pieces of code, referred to as modules: (1) Data loading and nowcasting, (2) Reproduction number estimates, (3) Vaccination coverage forecasting, (4) Incidence model, and (5) Care path model. The calculations done in modules 2 and 3 rely only on the loaded data and user input, while modules 4 and 5 also build further on the results of each previous module (see Fig. 1). This hierarchical structure implies that any change to parameters in a module updates the outputs in all dependent modules. However, by guiding the user through the modules in sequential order, the number of required computations can be drastically reduced.

### Data structure and definitions

The model needs data on infection incidence, vaccine doses, bed occupancy in general wards and intensive care units, and a simulation window for which a forecast is required. Although we developed this model for hospitals in Germany, based on data provided by the Robert Koch Institute^11^ and the Deutsche Interdisziplinäre Vereinigung für Intensiv- und Notfallmedizin (DIVI)^12^, the model can be applied to any country that has the following data available (preferably if in remote-accessible, machine-readable format):

1. Incidence, $$I_{g}(t)$$
2. Administered vaccinations, $$V_{d,g}(t)$$
3. Bed occupancy on general wards (GW) $B_{GW,g}(t)$ and ICU $B_{ICU,g}(t),$

where *t* denotes the time in days since a reference date before the start of the pandemic, *g* denotes a geographical subdivision (in the case of Germany, this is a district, i.e., Landkreis or Stadtkreis), and *d* denotes the dose of vaccine administered (1st, 2nd, or 3rd/booster). Incidence and vaccination data are needed for each day of the period of reference, while merely the value at the start date of the forecast is needed for bed occupancy.

The user defines the catchment area of interest, selecting geographical areas from the available list ($K_{all}$), into the catchment set ($K_u$). After this, the input data is summed over the selected areas:

1 $$\begin{aligned} I(t)= & {} \sum _{g\in K_u} I_g(t), \end{aligned}$$

2 $$\begin{aligned} V_d(t)= & {} \sum _{g\in K_u} V_{d,g}(t), \end{aligned}$$

3 $$\begin{aligned} B_{GW}(t)= & {} \sum _{g\in K_u} B_{GW,g}(t), \end{aligned}$$

4 $$\begin{aligned} B_{ICU}(t)= & {} \sum _{g\in K_u} B_{ICU,g}(t). \end{aligned}$$

The start of the simulation is denoted as $T_S$, for which the incidence, number of vaccinations, and bed occupancy are taken from the (last data-point of the) data, the first forecasted day is thus $T_S+1$. The length of the forecast is defined as *L*; the last forecasted date is therefore given as $T_E = T_S+L$. The size of any class of individuals in the model is given in absolute numbers of individuals; thus $S(0) = N$, with *N* being the population size.

Because many of the parameters in the dashboard’s models are continuously distributed (as Exponential, Gamma, or Weibull), and the model uses discrete time steps, we need to discretise the parameter distributions. For each continuous distribution, with probability density function (PDF) $f_c(t)$, the discretised PDF is defined as $f_d(t) = \int _t^{t+1}f_c(x)dx.$

User input for all distributions is given in terms of the mean $\mu$ and standard deviation $\sigma$, and distribution parameters are then determined by moment matching. For a gamma distribution, this means that $f(x,\alpha ,\beta ) = f(x,\alpha =\mu ^2/\sigma ^2, \beta =\mu /\sigma ^2)$, for a Weibull distribution, $f(x, k, \lambda ) = f(x,k=(\frac{\sigma }{\mu })^{-1.086}, \lambda =\mu / \Gamma (1+1/k))$, and for an exponential distribution $f(x,\lambda )= f(x, \lambda=1/\mu ).$ All model parameters are listed in the attached Table S1 in the Supplementary Information.

The model can take the effect of emerging Variants of Concern (VoCs) into account, based on user input on the VoC proportion and increased transmissibility. For a full description of the implementation of VoC in the model see Supplementary Text S1.

### Vaccination model

The vaccination model consists of two parts. In the first, we forecast the number of people vaccinated over the length of the forecast for each of the doses (1st, 2nd, and 3rd/booster dose), while in the second part we convert these administered vaccinations into population-level protection against transmission.

The first doses ($V_{1}(t)$) are assumed to be administered at the same rate as during the last observed week:

5 $$\begin{aligned} V_{1}(t>T_{S}) = \sum _{i=T_S-6}^{T_S}V_{1}(i)/7. \end{aligned}$$

The second dose is assumed to be administered at a fixed time delay after the first dose. This time delay $\Delta T_V$ is extracted from the data as the maximum time for which

6 $$\begin{aligned} V_{1}(T_S-\Delta T_V) \ge V_{2}(T_S), \end{aligned}$$

holds. The second doses are then a direct reflection of the number of first doses $\Delta T_V$ days ago,

7 $$\begin{aligned} V_{2}(t) = V_{1}(t-\Delta T_V). \end{aligned}$$

The uptake of the second dose is thus assumed to be identical to the uptake of the first dose.

The daily administered third/booster doses are assumed to be a continuation of the mean daily third doses over the previous week:

8 $$\begin{aligned} V_{3}(t>T_{S}) = \sum _{i=T_S-6}^{T_S}V_{3}(i)/7. \end{aligned}$$

This is in line with the forecasting of the first doses, with the exception that the cumulative number of third doses is not allowed to exceed the cumulative number of second doses a certain delay ($\Delta T_B$) ago, such that

9 $$\begin{aligned} \sum _{i=0}^{T_S} V_{3}(i) \le \sum _{i=0}^{T_S-\Delta T_B} V_{2}(i), \end{aligned}$$

has to hold.

#### Population protection

Each dose is assumed to have a time-dependent additive effect on the population-wide protection against infection. At the individual level, $G_{d}(\tau )$ denotes the proportion of individuals no longer susceptible to infection $\tau$ days after the administration of dose *d*. This function thus serves to simulate the delay between vaccine administration and maximum vaccine protection at the individual level. We assume no waning of immunity, such that $G_d(\infty ) = E_d$, with $E_d$ the vaccine effectiveness against transmission elicited by doses *d*.

The additive effect on vaccine effectiveness of each additional dose is defined as ${E_d}^*= E_d-E_{d-1}$; (Consequently, ${E_2}^*= E_2-E_{1}$ and ${E_3}^*= E_3-E_{2} = E_3-({E_{2}^*}+{E_{1}})$). Consequently, the additive increase in an individual’s protection due to an extra dose can be written as:

10 $$\begin{aligned} {G_d}^*(\tau ) = G_d(\tau ) \frac{{E_d}^*}{E_d}. \end{aligned}$$

$G_d(\tau )$ is input as the cumulative distribution function (CDF) of a normal distribution with mean $\mu (G_1)=15$, $\mu (G_2)=15$, $\mu (G_3)=7$, and standard deviation $\sigma (G_1)=3.8$, $\sigma (G_2)=6.5$, $\sigma (G_3)=3.8$ as default values. The values were chosen to mimic a delay of slightly longer than 2 weeks for the first two doses, and 1 week for the booster (third dose), with more variation between individuals in the response against the second dose, as we lack precise estimates of these delays.

The population protection at time *t* is then given by,

11 $$\begin{aligned} G_p(t) = \sum _{d}^{\{1,2,3\}}\sum _{i=0}^{t-1} \left( {G_d}^*(t-i) \frac{V_{d}(i)}{N} \right) . \end{aligned}$$

which is used in both the R forecasting step and the incidence model.

### Estimation and forecasting of R

In order to inform the transmission process part of the incidence model, we need to forecast the reproduction number of the pathogen over the model timeframe. This forecast is based on the observed time-varying effective reproduction number ($R_e(t)$), and implemented either as the static continuation of the most recent observation, or forecasted using an ETS (Error, Trend, Seasonal) model^13^ based on the last 100 days of observations, depending on the user’s preference. Furthermore, we provide the option to incorporate the effect of a Variant of Concern (VoC) on the development of the $R_0(t)$. In all cases, we use estimated values of $R_e(t)$ from the available data up to the simulation start date $T_S$, $R_e(t\le T_S)$, and forecasted values after the simulation start date ($R_e(t>T_S)$).

We estimate the time-varying effective reproduction number $R_e(t)$ using the Cori et al. R estimation method^14^ as implemented in the EpiEstim R package (version 2.2-4), with a given non-parametric Serial Interval (SI). This SI distribution denotes the number of days between equal disease events (e.g., onset of symptoms) of directly connected cases. The default SI is assumed to be Gamma distributed with a mean of 5 days and a standard deviation of 4.9. These values were chosen to reflect estimates of mean SI from multiple studies^15^ with a relatively high standard deviation.

By keeping track of the cumulative number of infected individuals, as well as the previously calculated population protection ($G_P(t)$), we can calculate $R_0(t)$ from $R_e(t)$, given that $R_e(t)$ is related to $R_0$ through the number of susceptibles at time *t*, defined as *S*(*t*):

12 $$\begin{aligned} S(t)= & {} N (1 - \sum _i^t \frac{I(i)}{N}) (1-G_P(t)) \end{aligned}$$

13 $$\begin{aligned} R_e(t)= & {} R_0(t) \frac{S(t)}{N}, \Rightarrow R_0(t) = \frac{R_e(t) N}{S(t)}. \end{aligned}$$

#### ETS model

To produce a dynamic forecast of $R_0(t)$, we used an ETS model which underlies an exponential smoothing method^13^. We use the ETS(A,A,N) model, which has additive errors (A), additive trend (A) and no seasonality (N); We use a $\log (R_0(t))$ time series of 100 days, i.e. $\log (R_0(T_S-99... T_S))$, as input for the ETS model. The logarithm serves to forecast the relative changes in $R_0$ and avoids negative $R_0$ forecasts. Per model iteration, we pick a random quantile for the prediction interval and use this as the trajectory for $R_0(t>T_S)$. A detailed description of the ETS model is given in the Supplementary Information Section S2.

### Incidence model

The incidence forecast model is based on a stochastic implementation of a Susceptible-Infected-Recovered (SIR) model. The model describes the number of new cases as a function of the population still susceptible (*S*(*t*)) to infection and of infection pressure (*P*(*t*)) exerted by all those currently infectious, and the total population size (*N*). The size of the components is given as absolute numbers of individuals; thus $S(0) = N$. The infection process is governed by the forecasted basic reproduction number $R_0(t\ge T_S)$ computed in the preceding modules of the framework.

If no VoC is defined, the model describes the dynamics of a single variant (the background variant). Conversely, the model converts into a two-strain model if a VoC is defined, keeping track of the infected individuals for either variant as well as the total number of individuals susceptible to either variant.

The size of the susceptible class is determined by both the cumulative number of infected individuals and the population level protection through vaccination, as defined before,

14 $$\begin{aligned} S(t) = N (1-G_P(t))\left( 1- \sum _{i=0}^{t}\frac{ I(i)}{N}\right) \end{aligned}$$

The infection pressure exerted on *S*(*t*) is then given as the weighted number of infected individuals in the preceding serial interval,

15 $$\begin{aligned} P(t) = \sum _{i=0}^t H(i)I(t-i) \end{aligned}$$

where *H*(*i*) is the serial interval distribution, defined as the probability that the proportion of cases causing new cases *i* days after they were reported themselves. These are thus all past cases that are able to cause new cases on the current day *t*, weighted by the serial interval distribution. Given the current $R_0(t)$ and *P*(*t*), the expected mean total number of newly infected individuals on day t+1 is $\overline{I}(t+1)=R_0(t) P(t)$.

The transmission rate per infected individual (The probability of infecting each of the other individuals in the population), is given as $\beta (t) = R_0(t)/N$.

The infection pressure exerted on each individual within the population at time *t* is then calculated as

16 $$\begin{aligned} P_I(t)= 1- (1-\beta (t))^{P(t)}, \end{aligned}$$

which for low numbers of infected individuals (and therefore low *P*(*t*)) can be simplified to $P_I(t)= \beta P(t).$ The newly infected individuals ($I(t+1)$) are then randomly picked from a binomial distribution $I(t+1)=Binom(p=P_I(t),N=S(t))$.

### Care path (Within-hospital) model

To forecast bed occupancy in the hospital, the main endpoint of this work, we first simulate the path of each single patient admitted to the hospital through the general ward, ICU, and step-down units (care path model), as described in Donker et al.^16^. Then we join this model with the predicted incidence to forecast the hospital admission rate and the bed occupancy.

We use the following parameters to model the care path of individual patients through the hospital.

- Length of stay (LoS) distribution on general wards (GW),
- LoS distribution on intensive care units (ICU),
- LoS distribution on step-down units (SDU),
- Proportion of patients being transferred from GW to ICU,
- Proportion of patients being transferred from ICU to SDU.

It is possible to add the proportion of patients directly admitted to ICU ($H_I$) to this list, but this is usually estimated from the data (see below).

Once all admissions and discharges are determined, we track the total number of patients on each ward type on each day of the forecast, presenting the final result of the forecast. Note that, despite modelling the general ward and step-down unit separately, we combine both wards into a single number of beds occupied on the general ward in any other step, because the occupied beds in both cases are usually reported as part of the same type (i.e., occupied non-ICU beds).

#### Admission rate

To estimate the proportion of reported cases being admitted to hospital, we need to compare the incidence of reported cases to the total number of patients in hospital at a given moment (prevalence). This can be achieved by taking the length of stay of admitted patients into account. We create a complete Length of Stay distribution $L(t_a)$, defined as the proportion of patients still in hospital $t_a$ days after admission by simulating the care paths of a large number of patients ($N_P=10000$) with the same admission date using these parameters. For each day after admission, we then track how many patients are still in hospital ($N_H(t_a)$), resulting in the Length of Stay distribution,

17 $$\begin{aligned} L(t_a)=\frac{N_H(t_a)}{N_P} \end{aligned}$$

The admission rate can then be directly estimated using the current number of occupied beds in general wards $B_{GW}$ and in ICU $B_{ICU}$

18 $$\begin{aligned} \alpha (t) =\frac{B_{GW}(t)+B_{ICU}(t)}{ \sum _{i=0}^{t} I(i) L(t-i) }, \end{aligned}$$

which we assume to remain stable after the start of the simulation: $\alpha (t>T_S) = \alpha (T_S)$).

The same method delivers the distribution of admitted patients on the GW $L_{GW}(t_a)$ and on the ICU $L_{ICU}(t_a)$. Note that $L(t_a) = L_{GW}(t_a)+L_{ICU}(t_a)$, and while $L(0)=1$, $L_{GW}(0) \le 1$ and $L_{ICU}(0) \le 1$. To be exact, $L_{ICU}(0) = H_I$, reflecting the directly to ICU admitted patients, and $L_{GW}(0) = 1- H_I$,

The admission rate can also be calculated for the separate wards,

19 $$\begin{aligned} \alpha _{GW}(t)= & {} \frac{B_{GW}(t)}{ \sum _{i=0}^{t} I(i) L_{GW}(t-i) }, \end{aligned}$$

20 $$\begin{aligned} \alpha _{ICU}(t)= & {} \frac{B_{ICU}(t)}{ \sum _{i=0}^{t} I(i) L_{ICU}(t-i) }. \end{aligned}$$

We can calculate the expected number of ICU beds under the assumption that no patients enter the ICU directly.

21 $$\begin{aligned} {B^*_{ICU}}(t)=\left( B_{GW}(t)+B_{ICU}(t)\right) \frac{ \sum _{i=0}^{t} I(i) L_{ICU}(t-i)}{\sum _{i=0}^{t} I(i) L(t-i) }. \end{aligned}$$

The surplus of ICU patients ($B_{ICU}(t)-{B^*_{ICU}}(t)$) is then caused by the direct admission of patients to ICU, and calculated as the ICU surplus over the total number of occupied beds.

22 $$\begin{aligned} H_I(t) = \frac{B_{ICU}(t)-{B^*_{ICU}}(t)}{B_{GW}(t)+B_{ICU}(t)} \end{aligned}$$

Similar to $\alpha (t)$, we assume that $\alpha _{GW}(t)$, $\alpha _{ICU}(t)$, and $H_I(t)$ remain stable after $T_S$.

This admission rate then determines the number of individuals admitted to the hospital, entering the care path model, pulled from the binomial distribution

23 $$\begin{aligned} Binom(p=\alpha ^*(t),N=I(t)). \end{aligned}$$

The care path model then depends on determining lengths of stays on each ward and movements between ward types for each individual admitted patient sampled from their respective distributions.

However, the care path model needs to determine the lengths of stays for the patients already in hospital at $T_S$, that is $B_{GW}(T_S)$ and $B_{ICU}(T_S)$. To simulate their future discharge and transfer events, we create an admission record using a uniform number of patients per day for the 100 days preceding $T_S$ and simulate their care paths.

We then select all patients present in the GW or ICU at $T_S$, creating the “Current” patient population in the hospital. From the current population, we randomly select $N_{GW} = B_{GW}(T_S)$ and $N_{ICU} = B_{ICU}(T_S)$ patients to recreate the discharges for the current population.

### Dashboard server implementation

The main backbone of the model is written in R (version 4.1.2^17^) as an R-Shiny^18^ (version 1.6) app running using shiny-server on an 8-core, 16GB ram, Ubuntu (version 20.04.3) VM server. An IP-hashed load balancer divides traffic over eight separate instances of shiny-server, reducing the number of concurrent users per shiny-server. We measure the performance of the model based on this infrastructure.

The most computationally demanding part of the care path model was written in the Julia programming language^19^ to improve performance. Julia is rapidly gaining momentum as a tool for scientific computing due to higher performance compared to languages like R or Python without compromising ease of use. The care path model written in Julia takes approximately 1 hundredth of the processing time of the R equivalent. We use JuliaConnectoR (v.1.0.0.9009)^20^ to allow communication between the Julia and R code bases. One of the drawbacks of Julia is that the functions require just-in-time compilation the first time they are used; the overhead related to compilation time exceeds the running time of the entire care path model. To address this issue, we generated a fully compiled version of the model code^21^, virtually eliminating loading time. Once the main server is started, a concurrent Julia session is created and is shared between all shiny-server sessions. For a detailed dashboard performance benchmarking, see Supplementary Information Section S3.

### User interface

**Figure 2:**
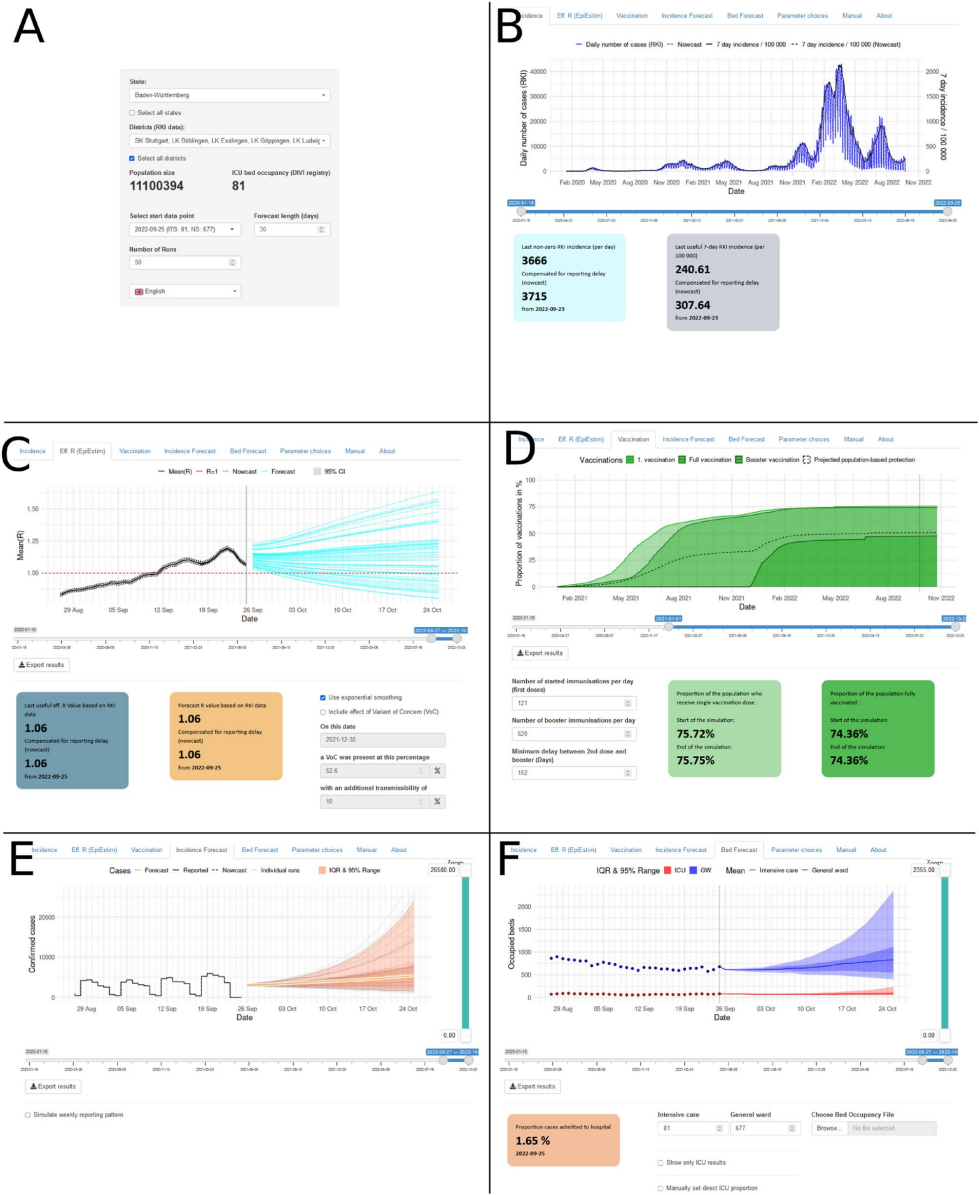
The user interface of the on-request COVID-19 bed demand forecasting model. (**A**) Side panel with basic controls, (**B**) Reported incidence, (**C**) Effective R forecast, (**D**) Vaccination forecast, (**E**) Incidence forecast, and (**F**) Bed occupancy forecast.

The user interface of the dashboard is shown in Fig. 2. It is divided into sections (tabs) following the main modules of the model, in combination with a side panel showing the basic controls: the catchment area selection, the forecast start date, the number of simulation runs, and the simulation length in days. On each tab, parameter choices can be made that affect the current and further tabs, but not any previous tabs. In this way, we prevent unnecessary re-running of the simulations.

Tabs are ordered as follows: (1) Incidence, showing the daily reported number of cases with and without the nowcast. (2) Vaccination, showing the cumulative number of vaccine doses administered, as well as the forecast of vaccinations. It also includes the option to change the assumptions on the future vaccinations (number of first doses and booster doses per day, and the minimum delay between 2nd dose and booster). (3) Effective *R*, showing the time-varying *R* estimation based on the EpiEstim package. This tab also includes the option to use the ETS model and the option to include a variant of concern (VoC), together with the needed VoC parameters. (4) Incidence forecast, showing the results of the incidence model, with the option to include the reporting pattern related to the weekdays. (5) Bed forecast, reporting the results of the within-hospital care path model. The model is run using data on the current number of occupied beds in the hospital of interest, inputted either manually or by uploading a specifically structured file. (6) Parameter choices. This tab includes all parameters included as default values in the other tabs, which should only be changed by users with more advanced knowledge of the underlying model.

### Forecast validation

**Figure 3:**
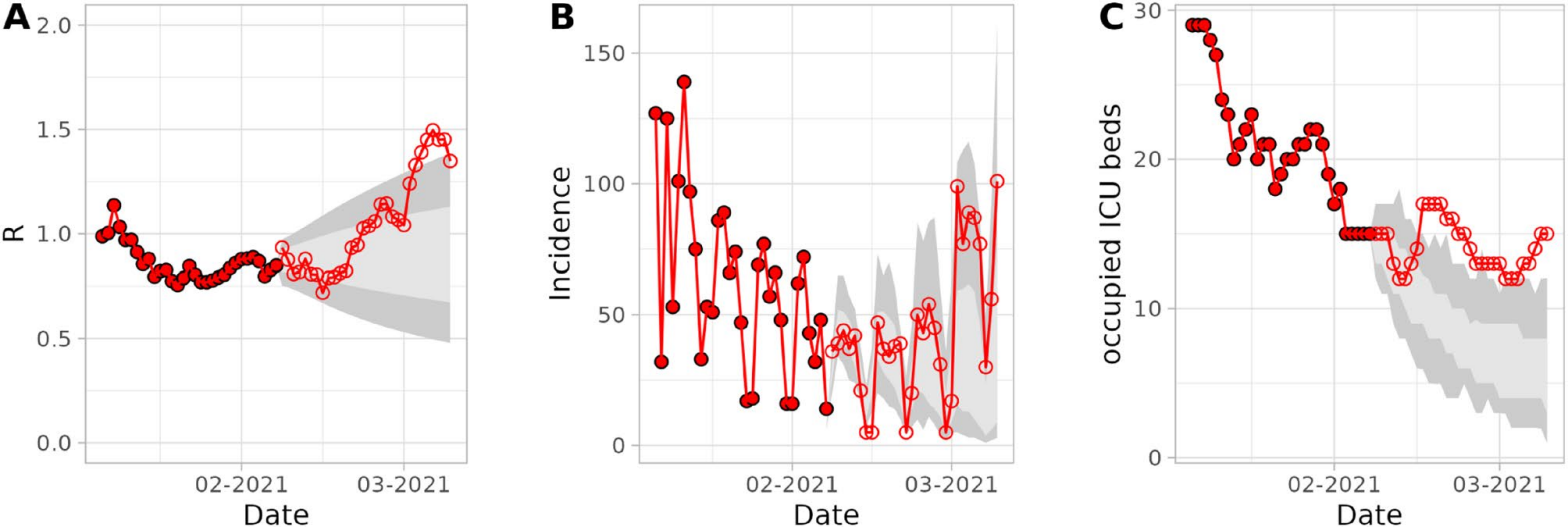
Example forecasts of $R_t$, Incidence, and occupied ICU beds. The forecasts start on the 07-02-2021. Filled red dots represent the 30 previous days and empty red circles represent 30 forecasted days. The performance of the forecasts was validated by calculating its accuracy and precision. Accuracy is defined as the number of observations (dots) falling into the interquartile range (IQR, light grey) and the 95% interval range (dark grey). In this example, the accuracy of the $R_t$ forecast is 0.43 and 0.2, respectively.

Figure 3 shows example forecasts of $R_e(t)$, incidence, and bed occupancy. The performance of the model was assessed based on the accuracy and precision of the forecasts from 470 different starting days. The accuracy is defined by determining the number of observed values at day *d* (with $d=1$ being the start of the simulation) that fall within the interquartile range (IQR) or within the 95% range of the forecast simulation distribution divided by $N_d$. $N_d$ is defined as the total number of observed values at day *d* in all 470 forecasts, or in other words, the number of starting dates for which we have data *d* days into the future:

24 $$\begin{aligned} accuracy(d) = \frac{\sum _{i}^{N=470} {obsw_{id}}}{N_d} \end{aligned}$$

where $obsw = 1$ if the observed value at day *d* lies within the range (if not, $obsw = 0$). The IQR and 95% range of each forecast are defined by their 100 independent model runs. The day *d* goes from 1...30 with $d = 1$ being the first forecasted day. Similarly, the precision was defined as the size of the IQR divided by the maximum forecast value at day *d* averaged over all 470 forecasts with *max* being the maximum forecasted value from all simulation runs for forecast *i* at a given day *d*:

25 $$\begin{aligned} precision(d) = \frac{\sum _{i}^{N=470} (IQR/max)_{i,d}}{N_d} \end{aligned}$$

To evaluate the time dependency of the performance of the forecasts we analysed the mean absolute scaled error (MASE)^22^ (see Supplementary Material section S6). Further, we calculated the bias of the forecast to determine if our method results in a systematic over- or underestimation (see Supplementary Material Section S6).

### Hospital-specific forecasts

We forecasted the bed demand for four university hospitals in Baden-Württemberg, Germany, located in Freiburg, Mannheim, Heidelberg, and Tübingen. Participants from each hospital provided a list of counties they perceived as their main catchment area, as well as a list of the number of occupied beds in both ICUs and General wards between 14 October 2020 and 27 January 2022. Supplementary Table S3 lists the counties in the specified local catchment areas and their total population. In addition to those hospital-specific catchments, we produced forecasts based on every German state as an individual catchment, plus the whole of Germany (all states combined). We created bed demand forecasts for each hospital starting at every day between 14-10-2020 and 27-01-2022, resulting in a total of 470 forecasts. For each of the 470 forecasts we performed 100 independent model runs, with a forecasting length of 30 days. The generated forecasts were compared to the actual observed bed occupancy data. We tested the effect of forecasting $R_e(t)$ on the bed forecast using either the exponential smoothing (ETS) or the naive $R_e(t)$ forecast, as well as including or excluding the added fitness advantage of a given VoC to the prediction. Additionally, a scenario was tested where the selected catchment and the area corresponding to the observed hospital have a similar population size but do not overlap in their possible admitted patients (e.g. a city which is geographically far away from the hospital of interest). To accomplish this, we chose representative catchments around the city of Rostock, Germany and matched their bed occupancy to the catchments of the four investigated hospitals.

## Results

In order to assess the performance of the model, we measured its accuracy and precision in term of distribution of forecasted values *d* days into the forecast from the starting day, over all starting days (See Fig. 3). Precision is then defined as the mean of the normalised inter-quartile range sizes, while accuracy is defined as the proportion of forecasts where the observed value falls within a defined range. Note that by definition, if the forecasted distribution is consistent with the real distribution, 50% of the forecasted observations are expected to lie within the interquartile range, while 95% of observed values should lie within the 95% range. Obtaining a resulting accuracy as close to the defined range as possible, with the lowest possible forecast distribution range size is therefore the set goal.

### Forecast of the $\varvec{R_e(t)}$-value

The forecasts of the $R_e(t)$ value for the Freiburg-specific catchment areas (see Supplementary Fig. S3 , performed poorly when using the naive model, with the accuracy dropping to a value of  12.5% on day 10. Using the ETS model greatly improved the forecasts, with very high accuracy at the first days of the forecast which declined to 50% at day 5, and slightly increased again from day 15 onwards. Analogically, around 90% of $R_e(t)$-values lie within the 95% range of $R_e(t)$ forecasts using the ETS model. Including the Variant of Concern in the calculation does not significantly affect the forecast’s accuracy (see Supplementary Fig. S3). Long-term accuracy (day 30) was generally highest for smaller catchment sizes, with the local catchment outperforming the other catchments. At the same time, the precision of the forecast remains constant when using the naive method, by definition, because the forecasted $R_e(t)$ value does not change over time (see Supplementary Fig. S4). The ETS model shows a clear reduction in precision over time, with smaller catchment areas resulting in the lowest 30-day precision. Inclusion of the VoC has no effect on the forecast’s precision.

### Incidence

**Figure 4:**
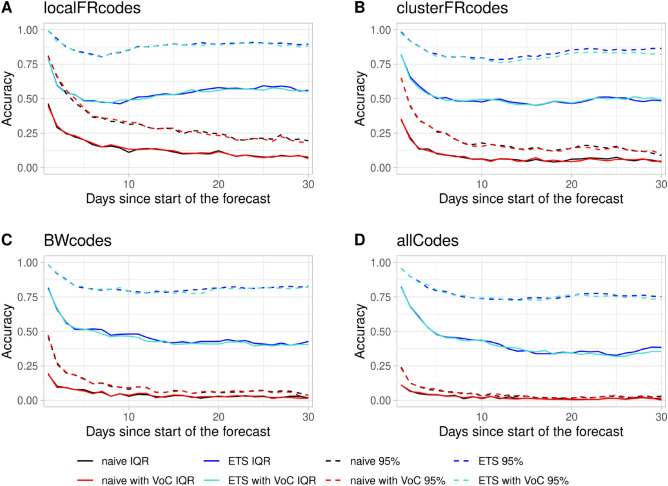
Accuracy of the Incidence forecast. Accuracy is shown for the (**A**) local Freiburg catchment, (**B**) the Freiburg cluster, (**C**) Baden-Württemberg, (**D**) and whole Germany over 30 days. Solid lines represent accuracy based on the interquartile range (IQR) while dashed lines represent accuracy based on the 95% range. The $R_t$-value was predicted using the naive forecast (black lines), naive forecast including VoC (red lines), exponential smoothing (ETS) (dark blue lines), and exponential smoothing including VoC (turquoise lines).

**Figure 5:**
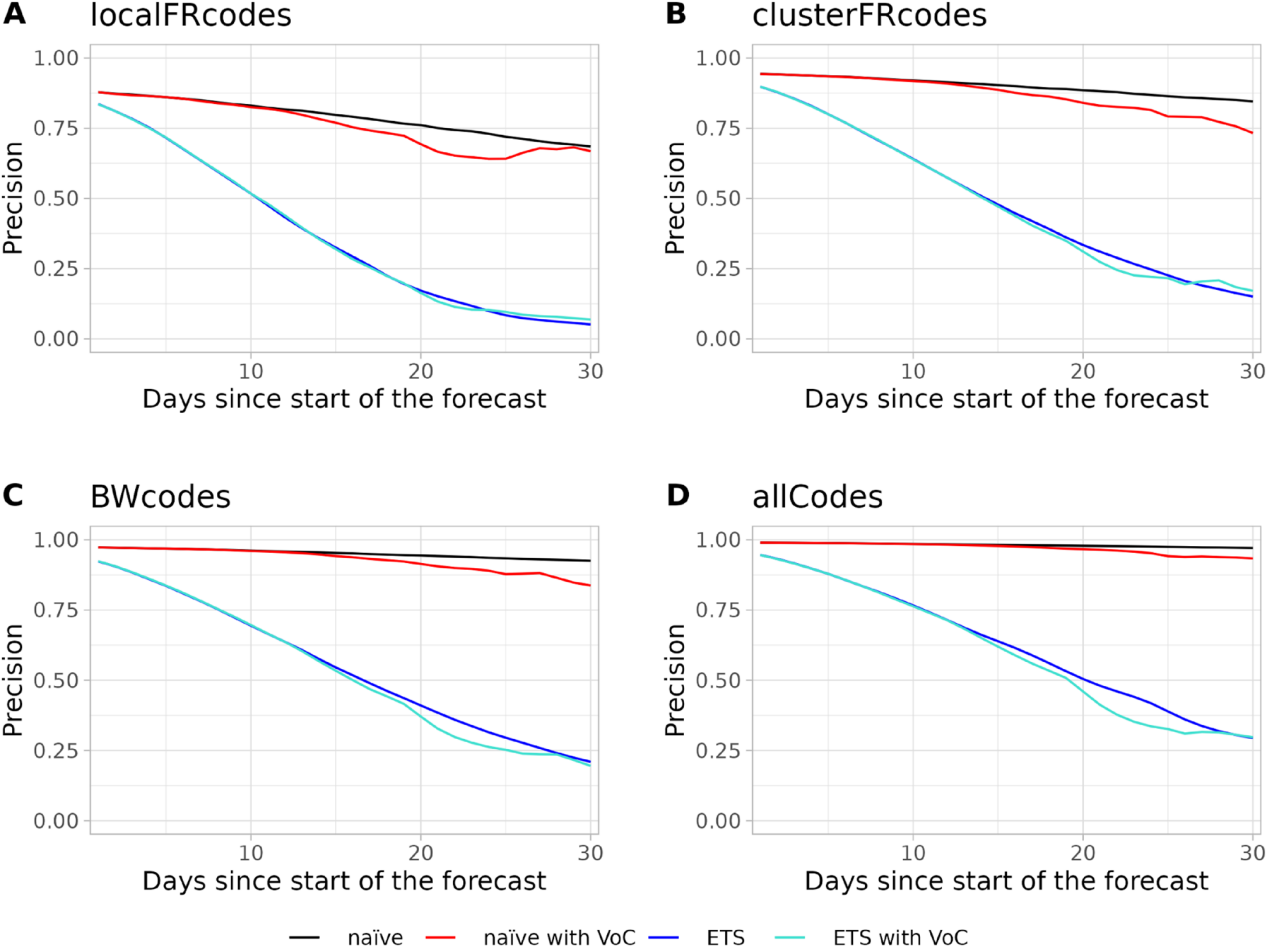
Precision of the Incidence forecast. Precision is shown for (**A**) local Freiburg catchment, (**B**) the Freiburg cluster, (**C**) Baden-Württemberg, (**D**) and whole Germany over 30 days. The $R_t$-value was predicted using the naive forecast (black line), naive forecast including VoC (red line), exponential smoothing (ETS) (blue line), and exponential smoothing including VoC (turquoise line).

The accuracy of the incidence forecast for the local Freiburg catchment increases with the days since the start of the forecast when using the ETS model. The IQR based accuracy lies below 50% for the first 15 days of the forecast and remains at 50% for the rest of the forecast (Fig. 4). However, the accuracy based on the naive method for the IQR does not surpass 25%. Similar to $R_e(t)$, increasing the population size of the catchment leads to a reduction of accuracy and an increase in precision of the incidence forecast for both the IQR and 95% confidence range (Figs. 4 and 5). For all catchments the accuracy of the forecast improves over time, this is however at the cost of the precision as the interquartile range widens.

### Bed forecast based on bed occupancy of the university hospital of Freiburg

**Figure 6:**
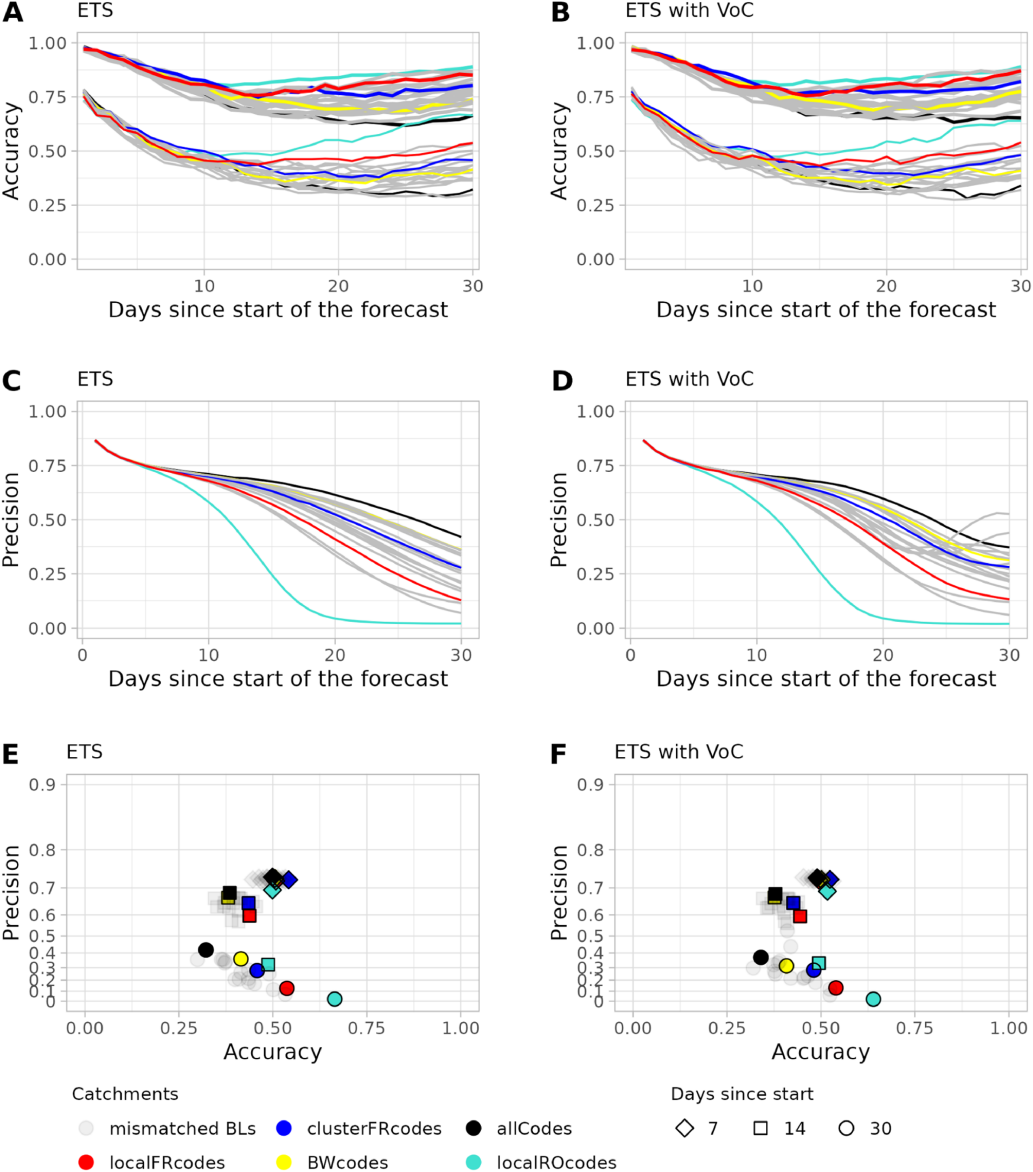
ICU bed forecast based on the bed occupancy of the University Hospital in Freiburg. Forecasts are based on different methods for predicting the $R_t$-value: exponential smoothing (ETS, left vertical panel) and exponential smoothing including VoC (right vertical panel). The first horizontal panel (**A**,**B**) shows the accuracy. Colours represent different catchment areas (red: Freiburg local, blue: Freiburg cluster, yellow: Baden-Württemberg (BW), turquoise: Rostock, black: whole Germany, grey: all states separately except BW). The lower bundle of lines represent accuracy based on the interquartile range (IQR) while the upper bundle represent accuracy based on the 95% confidence range. Second vertical panel (**C**,**D**) depicts relative precision. Third horizontal panel (**E**,**F**) shows precision on a log transformed scale versus accuracy. Diamond shapes represent the 7th day, square shapes the 14th day, and circles the 30th day of the forecasts.

For the bed forecast for the university hospital of Freiburg, we observe a steady decline in accuracy for about 10 days, with a slight increase in accuracy after day 14 (Fig. 6A,B) when using the ETS model regardless if a VoC is included or not. In contrast, applying the naive model only leads to a steady decline in accuracy (see Supplementary Fig. S6). For the four main choices of catchment areas, the local catchments are more accurate than the larger catchment areas: Highest accuracy is obtained using only the three local Landkreise, followed by the FR cluster, Baden-Württemberg, and ending with Germany as a whole. For precision (Fig. 6C,D) we observe a similar effect as for accuracy; precision is high at first, and declines until day 7. After that, the precision starts to depend increasingly on the size of the catchment population. This dependency is more pronounced for forecasts including exponential smoothing. The precision is lowest for the local Freiburg catchment and increases for the Freiburg cluster and Baden-Württemberg with being highest for Germany as a whole. While most forecasts steadily decrease in precision, for certain catchments the precision of forecasts including the VoC fluctuates towards later forecast dates, first decreasing then increasing again.

### Different catchments

When comparing different catchment areas (Fig. 6A,B), the smaller hospital-specific catchments produce the most accurate, but least precise, results. However, at the level of states (Bundesländer), Baden-Württemberg only seems to produce forecasts that are not more accurate than those based on other (incorrect) states. At the same time, the forecasts based on Baden-Württemberg are more precise (Fig. 6C,D) than most of the forecasts based on the other states. The forecast for the Freiburg hospital based on a small, but mismatched, catchment area (the Rostock catchment) shows a higher accuracy than the one based on Freiburg-specific local catchment (Fig. 6A,B). This high accuracy, however, comes at the cost of precision. After 10 days the accuracy for the local Rostock area bed forecast with ETS is the highest in comparison to all other catchments, while the precision for this area is the lowest, approaching 0 after 20 days (Fig. 6C,D). A high accuracy (> 60% for IQR) and low precision for the Rostock catchment was also observed for the $R_e(t)$-value forecast (see Supplementary Fig. S16) and the incidence forecast (see Supplementary Fig. S17). When plotting accuracy against precision for ICU beds (Fig. 6E,F) at days 7, 14, and 30, we observe that predictions made with larger catchment areas deliver higher precision with lower accuracy, and smaller catchments vice-versa. In general, the Freiburg-specific catchments are present in the top-right of the clouds of results based on mismatched catchment areas: they deliver a good match between precision and accuracy. The inclusion of VoC to the exponential smoothing has only a marginal effect on precision and accuracy.

### Bed forecast based on bed occupancy of university hospitals of Freiburg, Mannheim, Heidelberg, and Tübingen

**Figure 7:**
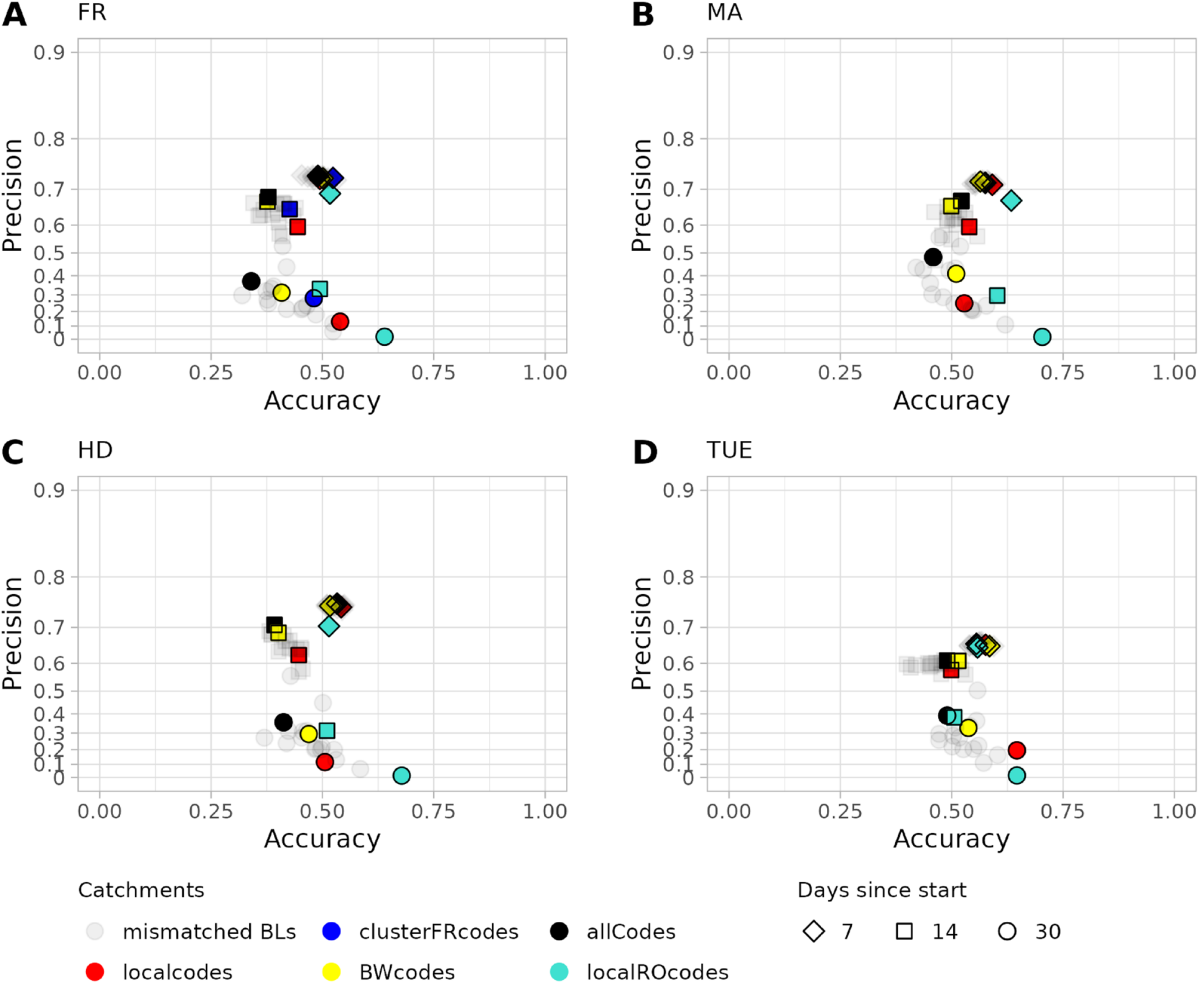
Precision versus Accuracy of ICU bed forecasts. Results are based on the bed occupancy of the University Hospitals in (**A**) Freiburg (FR), (**B**) Mannheim (MA), (**C**) Heidelberg (HD), (**D**) Tübingen (TUE). Forecasts are based on the $R_t$-value predicted by exponential smoothing including VoC (ETS with VoC). Colours represent different catchment areas (red: local, blue: Freiburg cluster (only in FR plot), yellow: Baden-Württemberg (BW), turquoise: Rostock, black: whole Germany, grey: all states separately except BW). Diamond shapes represent the 7th day, square shapes the 14th day, and circles the 30th day of the forecasts.

Interestingly, the forecast based on bed occupancy of the university hospital in Mannheim has a better accuracy but slightly lower precision at day 7 for all catchments as compared to forecasts based on Freiburg and Heidelberg (Fig. 7A,B). The forecasts for Mannheim on day 14 and 30 perform better as compared to Freiburg as both accuracy and precision increase. Overall, the forecasts for Freiburg, Mannheim, and Heidelberg follow the same observed trend where at day 30 larger catchments lead to increased precision but lower accuracy regardless if a VoC is included into the calculation (Fig. 7A–C, Figs. S7–S9). Similar to forecasts for local FR catchments, forecasts based on Mannheim and Heidelberg bed occupancy for the matching local catchments and for Baden-Württemberg based on 7 and 14 days lie at the top right end of the cloud representing all other states, indicating that best forecast performance is obtained when the bed occupancy data matches the catchment (Fig. 7). Forecasts which are based on bed occupancy from the university hospital in Heidelberg perform similarly to forecasts based on occupancy data from Freiburg at day 7 and 14, while the accuracy at day 30 is higher with a similar precision. Forecasts for Mannheim have a higher accuracy but slightly lower precision than Freiburg and Heidelberg. Interestingly, forecasts for Tübingen are less precise than the ones for FR, MA, and HD but more accurate than for FR and HD (Fig. 7 and Supplementary Figs. S7–S9). This low precision might be explained by low bed occupancy over time which is observed for Tübingen (see Supplementary Fig. S5) Forecasts which are based on incidence of Rostock, representing a mismatched catchment with a similar population size as the hospital-specific catchments, leads to a significant decrease in precision especially later in the forecast for all chosen hospitals while increasing the accuracy for all hospitals except the one in Tübingen (Fig. 7). Forecast for general wards are overall more precise but less accurate than forecasts for intensive care units (see Supplementary Figs. S10–S13).

### Time dependency of the forecasts

To evaluate not only the mean performance of all forecasts but also the time-dependent forecast performance we computed the mean absolute scaled error (MASE) of the median of each set of forecast simulations corresponding to its start date for the given time period for the university hospital of Freiburg. MASE of the $R_e(t)$ forecasts peaks at days where there are turning points in the observed $R_e(t)$, indicating poor performance at those days (see Supplementary Fig. S14). This is expected behaviour for many forecasting models, and especially exponential smoothing can not predict the reversal of trends unless the tipping points follow a seasonality. When including the VoC a high peak can be observed at 12.01.2021 which corresponds to a steep decline of $R_e(t)$. A possible explanation is that at the start of the emergence of the Alpha variant its relative fitness advantage is highly overestimated (see Supplementary Fig. S1) leading to overestimation of the $R_e(t)$ forecast. The performance of bed forecasts is less dependent on turning points in the observed bed occupancy than the performance of the $R_e(t)$ forecasts and is improving with increasing catchment size (see Supplementary Fig. S15). MASE shows a number of peaks which are only present when VoC is included into the calculation. The peaks in MASE correspond to peaks observed in the bed occupancy forecast and disappear when the catchment size is increased (see Supplementary Fig. S15). To evaluate if there is any over- or underestimation in the local Freiburg catchment forecast we computed the Bias of the bed occupancy forecast over time. By definition, positive Bias represents an overestimation of values in the forecast. The highest peaks in the Bias are usually positive, meaning that in cases where the bed occupancy is overestimated this overestimation is usually high (see Supplementary Fig. S14). Nevertheless, the median of the Bias is small but negative (-2.1 beds) indicating a slight tendency for underestimating bed occupancy.

## Conclusion

We show the importance of employing local data rather than national data when producing epidemic forecasts. Forecasts of bed demand produced based on the incidence in the specific catchment area of the hospital of interest, better predicted the local future bed demand than those based on the incidence of the entire country or state. Such hospital-specific bed demand forecasts can help inform hospital planning, e.g. by cancellation of elective surgeries. This has the potential to reduce the need to transfer critically ill patients between hospitals, because the local bed capacity can be better adjusted to the local bed demand. Ideally, the forecast horizon is far enough for the adjustments to take effect but near enough to still be accurate and precise enough to be informative. We observe a clear trade-off between the accuracy and precision of the forecast, governed by the size of the selected catchment area: larger catchment sizes generally produce more precise results at the expense of their accuracy, while smaller catchments result in more accurate but less precise forecasts. This is probably caused by a reduction of the stochastic variation in the underlying data. Basing the time-varying reproduction number ($R_e(t)$) value estimation on a larger catchment area (i.e. larger population size) reduces the fluctuations in the $R_e(t)$-time series which leads to a narrowing of the interquartile range of the forecasted distribution, increasing precision. However, at the same time, a larger proportion of the actual future bed demand then falls outside the forecast ranges, because the increased precision poorly reflects the uncertainty of the forecast. Correctly matched catchment areas seem to give the best trade-off between precision and accuracy. Surprisingly, some forecasts based on states far away from the hospital of interest produced more accurate results than the “correct” state, Baden-Württemberg in this case, but only because their precision is very low. In case of a smaller mismatched catchment area, in this case the Rostock catchment area, high fluctuations in the $R_e(t)$-value lead to a wide IQR resulting in high accuracy but at the cost of precision. This shows that the number of hospital admissions roughly follows the incidence in the catchment population specific to the hospital in the short term. The ideal catchment to base forecasts on should thus reflect the true area from which the hospital usually admits patients, but should not be chosen too small to avoid loss of precision. Prior to predicting the hospital bed demand a number of steps have to be undertaken, including estimating and forecasting the $R_e(t)$-value and the incidence. Previous studies suggest that while the SIR model is relatively successful at predicting the number of COVID-19 cases at short time periods (< 3 months) it fails to do so for longer time periods^23^. One possible explanation is the use of a static $R_e(t)$-value. Our results show that using a dynamic $R_e(t)$-value greatly improves the incidence forecast as compared to using a single $R_e(t)$-value regardless of the catchment population size.

As the epidemic trajectory greatly depends on the transmission rate of the current variant we included VoCs in our model for periods when a new variant is taking over a previously predominant one. During the analysed period one of the three strains Alpha, Delta, or Omicron have been taken over the previous one in a total of 33% of the time. During this time a considerable change in the spreading dynamic could occur. In our analysis the inclusion of VoCs does not show any significant effect on $R_e(t)$, incidence, or bed forecast. A possible reason might be that the amount of days where a variant takes over another variant is relatively small compared to the total analysed period which would neglect the effect of a variant of concern, especially if the effect is small. Another reason is that the change in trajectory caused by a VoC is already implicitly taken into account from the preceding values when predicting $R_e(t)$. When considering forecasts of $R_e(t)$ on early days where a VoC is emerging, a yet not well established relative fitness advantage of the VoC can lead to $R_e(t)$ forecasts that are strongly off. For a small number of days those fluctuations also result in very high peaks in the MASE of the bed occupancy forecast. Those few high peaks are usually an overestimation of the bed occupancy.

Our analysis reiterates the importance of producing forecasts based on local data and knowledge. We show that the challenges surrounding such hospital-specific forecasting can be overcome by using a centrally developed model, producing forecasts on-request. This way, each hospital was able to produce bed demand forecasts based on their own local data and knowledge, while using the same underlying mathematical model. Such an implementation requires careful IT-planning and programming, to make sure the app is scalable and remains responsive at higher demands.

Forecasting models have, of course, their limits and limitations. First and foremost, they use the current situation to project the incidence and bed occupancy forward under the assumption that the epidemic situation remains the same. This means that a strongly growing epidemic will be expected to grow further unabated. In reality, the epidemic growth is strongly influenced by changes in contact patterns within the population, such as caused by non-pharmaceutical interventions (NPIs) and general risk perception in the community. Likely, a high incidence situation will trigger behavioural changes that reduce the peak size of the epidemic, or conversely, low incidence may trigger relaxation of NPIs. Because the model doesn’t take these changes into account, it is often inaccurate around the inflection points of the epidemic curve. Furthermore, local forecasts are by design less precise than those based on national-level data, because the predictions are based on both lower case numbers and smaller base population sizes. This means that estimates of reproduction numbers have wider confidence bounds, and incidence, admission rates, and lengths of stays are stronger influenced by stochastic variation in the model. This sometimes results in forecasts that predict anything between an empty and fully overwhelmed hospital. Although these predictions may seem useless, they avoid a false sense of security and echo the true uncertainty of the situation (i.e. anything can happen). However, for most of the time, the forecasts show a clear trend that can form the basis for further strategic planning, such as cancellation of elective surgeries or opening of designated COVID-19 wards.

The dashboard was successfully used by local healthcare providers, hospitals, and healthcare policymakers to evaluate incidence and hospital bed occupancy in Germany during the 2020-2022 COVID-19 pandemic. We argue that on-request forecasts are much more helpful in informing stakeholders at a local level where health management decisions, such as cancelling elective surgeries, directly affect the bed capacity. This way, the pandemic or epidemic response can be driven in near real-time on the level where it matters most.

### Supplementary Information

Supplementary Information.

## Data Availability

The code used to implement the dashboard, in the version used to prepare this manuscript, is available at: https://github.com/QUPI-IUK/Bed-demand-forecast/releases/tag/v.0.5.6. Data for Germany will be downloaded by the dashboard itself.
