# Supplementary material for: Forecasting local hospital bed demand for COVID-19 using on-request simulations
Source: Sci Rep. 2023 Dec 3;13:21321. doi: 10.1038/s41598-023-48601-8 (PMC10694139; doi:10.1038/s41598-023-48601-8)
Supplement: Supplementary file 1 — Supplementary Information. [file 41598_2023_48601_MOESM1_ESM.pdf]

Supplementary Material

Model parameters

	Parameter	Source	Description
Catchment	K_{all}	Database	Set of all possible districts
	K_u	User	Set of selected districts
	N_g	Database	Population size of district g
	N	Database	Total population size of all selected districts
Incidence	$I_g(t)$	Database	Number of infected individuals reported on day t in district g
	$I(t)$	Derived	Number of infected individuals reported on day t in all selected districts
	$I_B(t)$	Derived	Number of individuals infected with the background variant on day t in all selected districts
	$I_V(t)$	Derived	Number of individuals infected with the variant of concern on day t in all selected districts
Susceptibles	$S(t)$	Derived	Total number of susceptible individuals at day t
	$S_B(t)$	Derived	Number of individuals susceptible to infection with the background variant on day t in all selected districts
	$S_V(t)$	Derived	Number of individuals susceptible to infection with the variant of concern on day t in all selected districts
Vaccination	$V_{d,g}(t)$	Database	Number of dth doses administered on day t in district g
	$V_d(t)$	Derived	Number of dth doses administered on day t in all selected districts
	E_d	User	Vaccine efficacy of the dth dose (Default: Dose 1: 25% dose 2: 50%, dose 3: 78%)
	$G_d(t)$	User	Delay function for vaccine effect. Proportion of cases on day t after administration of dose d protected to the maximum E_d. (Default: see Table S2)
	$G_P(t)$	Derived	Population vaccine-based protection against infection, proportion of the population not susceptible to infection
	η	User	Immune evasion
Transmission	$R_e(t)$	Derived	Time-varying effective reproduction number at day t
	$R_0(t)$	Derived	Basic reproduction number at day t

	$R_{0,B}(t)$	Derived	Basic reproduction number of the background viral population at day t
	$R_{0,V}(t)$	Derived	Basic reproduction number of the variant of concern at day t
	$\sigma(t)$	User	Serial interval distribution, giving the proportion of new cases arising t days after their infector (Default: 5 days)
	$\beta(t)$	Derived	Transmission rate
	$P(t)$	Derived	Infection pressure; Total number of past cases weighted by the serial interval
	$P_I(t)$	Derived	The infection pressure exerted on each individual within the population
	A	User	Added fitness advantage of the variant of concern
	A_η	Derived	Added fitness advantage of the variant of concern, through immune evasion
	$\rho_B(t)$	Derived	Backround viral population proportion among all cases
	$\rho_V(t)$	Derived	Variant of concern proportion among all cases
	r	Derived	Growth rate
Bed occupancy	$B_{GW,g}(t)$	Database	Number general ward beds occupied on day t in district g
	$B_{ICU,g}(t)$	Database	Number intensive care beds occupied on day t in district g
	$B_{GW}(t)$	Derived / User	Number general ward beds occupied on day t
	$B_{ICU}(t)$	Derived / User	Number intensive care beds occupied on day t
Care path model	$H_I(t)$	Derived	Proportion of all patients admitted to hospital directly admitted to the ICU
	$L(t)$	Derived	Proportion of cases still in hospital t days after admission
	$L_{GW}(t)$	Derived	Proportion of cases present on the General ward t days after admission to the hospital
	$L_{ICU}(t)$	Derived	Proportion of cases present on the Intensive Care Unit t days after admission to the hospital
	$\alpha(t)$	Derived	Proportion of all cases admitted to hospital (Admission rate)
	$\alpha_{GW}(t)$	Derived	Proportion of all cases admitted to the General Ward (Admission rate)
	$\alpha_{ICU}(t)$	Derived	Proportion of all cases admitted to the ICU (Admission rate)
	$X_B(t)$	Derived	Number of infected cases with the background variant with pre-existing immunity
	$X_V(t)$	Derived	Number of infected cases with the Variant of Concern with pre-existing immunity
	$X^*(t)$	Derived	Proportion of all infected cases with pre-existing immunity

	$U(t)$	Derived	Proportion of all individuals that have immunity through previous infection and/or vaccination
	$U_B(t)$	Derived	Proportion of all individuals that have immunity against the background viral population through previous infection and/or vaccination
	$U_V(t)$	Derived	Proportion of all individuals that have immunity against the VoC through previous infection
	π	Derived	Primary infection hospitalisation risk
	γ	User	Hospitalisation risk relative to primary infection (Default 0.1)
Time	T_S	User	Start date of the simulation
	ΔT_V	Derived	Time between first and second dose
	ΔT_B	User	Time between second and third dose (Default: 152 days)
	t_ρ	User	Reference date for the Variant of Concern

Table S1: **Model parameters used in the intermediate steps when producing the bed forecast.**

Delay function	Distribution	Mean	Standard deviation
First dose	Gamma	15 days	3.8
Second dose	Gamma	15 days	6.5
Third dose	Gamma	7 days	3.8

Table S2: **Delay distributions for immune protection after vaccination, for each dose of the vaccine ($G_d(t)$).**

Forecasted catchments

Catchment names	Included counties	Population
localFRcodes	SK Freiburg i.Breisgau, LK Breisgau-Hochschwarzwald, LK Emmendingen	661204
localFRcodes	SK Freiburg i.Breisgau, LK Breisgau-Hochschwarzwald, LK Emmendingen, LK Ortenaukreis, LK Rottweil, LK Schwarzwald-Baar-Kreis, LK Tuttlingen, LK Konstanz, LK Lrrach, LK Waldshut, LK Bodenseekreis	2488821
localMAcodes	SK Mannheim, LK Ludwigshafen	482911
localHDcodes	SK Heidelberg, LK Rhein-Neckar-Kreis	709840
localTUEcodes	LK Tbingen, LK Reutlingen, LK Freudenstadt	633955
localROcodes	SK Rostock, LK Rostock, LK Vorpommern-Rgen	649687

Table S3: **Catchments used to generate bed forecasts.** The catchment areas, corresponding population sizes and the designated names used throughout this publication are given. LK (Landkreis) stands for county and SK (Stadtkreis) stands for cities which are a single county.

S1 Variant of Concern implementation

To model the impact of new pathogen variants emerging in the population [1], we need to consider how a variant of concern (VoC) has an advantage over the background viral population. We considered two possible mechanisms: 1) through higher transmissibility, increasing the base $R_0(t)$, or 2) through immune evasion, increasing the number of susceptible individuals available to the VoC.

Increased transmissibility

In case of increased transmissibility, we increase the basic reproduction number of the background viral population $R_{0,B}(t)$ by the relative added fitness advantage A,

$$R_{0,V}(t) = (1 + A)R_{0,B}(t).$$

Note that the previously calculated $R_0(t)$ is the average of the background and VoC reproduction numbers weighted by the proportion of cases caused by either (respectively $\rho_B(t)$ and $\rho_V(t)$),

$$\begin{aligned} R_0(t) &= \rho_B(t)R_{0,B}(t) + \rho_V(t)R_{0,V}(t), \\ &= (1 - \rho_V(t))R_{0,B}(t) + \rho_V(t)(1 + A)R_{0,B}(t). \end{aligned}$$

We can calculate $R_{0,B}(t)$, used as the general reference reproduction number in the model, from $R_0(t)$ as

$$R_{0,B}(t) = \frac{R_0(t)}{(1 - \rho_V(t)) + \rho_V(t)(1 + A)},$$

or we can directly calculate this from the observed $R_e(t)$ value

$$R_{0,B}(t) = \frac{R_e(t)N}{((1 - \rho_V(t)) + \rho_V(t)(1 + A))\, S(t)}.$$

The proportion of cases caused by the VoC ($\rho_V(t)$) over time can be described with a sigmoidal function. A similar approach to estimating fitness advantages for new variants has been used by others [2]. This is done by assuming both the background population and the VoC have a static growth rate unaltered by added immunity through new cases (i.e., the relation between their growth rates is fixed over time). We can calculate these growth rates (r) as a function of R_0, using the mean serial interval (σ) [3]

$$r = \frac{\log(R_0)}{\sigma}.$$

This means that the number of infections from both background variants and the VoC can be described as respectively

$$I_B(t) = I_B(0)e^{\frac{\log\left(R_{0,B}\right)}{\sigma}t}$$

and

$$I_V(t) = I_V(0)e^{\frac{\log\left((1+A)R_{0,B}\right)}{\sigma}t}.$$

The proportion VoC is

$$\rho_V(t) = \frac{I_V(t)}{I_V(t) + I_B(t)} = \frac{1}{1 + \frac{I_B(t)}{I_V(t}},$$

which converts to

$$\rho_V(t) = \frac{1}{1 + \frac{I_B(0)e^{\frac{\log\left(R_{0,B}\right)}{\sigma}t}}{I_V(0)e^{\frac{\log\left((1+A)R_{0,B}\right)}{\sigma}t}}}.$$

By defining the total number of infected individuals as $I_T(0) = I_V(0) + I_B(0)$, and using $I_B(0) = (1 - \rho_V(0))I_T(0)$, we get

$$\rho_V(t) = \frac{1}{1 + \frac{1-\rho_V(0)}{\rho_V(0)} \frac{e^{\log\left(R_{0,B}\right)t/\sigma}}{e^{\log\left((1+A)R_{0,B}\right)t/\sigma}}},$$

rewriting to

$$\rho_V(t) = \frac{1}{1 + \frac{1-\rho_V(0)}{\rho_V(0)} e^{-\frac{\log(1+A)}{\sigma}t}}.$$

This shows that we only need the relative added fitness advantage (A) in combination with a starting proportion of the variant ($\rho_V(t = t_\rho)$) at a certain reference date (t_ρ) to describe the proportion VoC over time. In the dashboard code, the reference date for the proportion of VoC does not overlap with the starting date of the incidence, vaccination, and bed occupancy data. In other words, with $I(t_I = 0)$ and $\rho_V(t_\rho = 0)$, $t_I \neq t_\rho$, because $\rho_V(t_I = 0) = 0$, making a precise estimate of $\rho_V(t)$ impossible, or at least impractical. We, therefore, set t_ρ to a later time point at which the ρ_V is identifiable.

The added relative fitness advantage of the VoC can then be estimated from data about the proportion VoC among all isolates per week in the given catchment area, available through for instance genomic surveillance efforts. The user can then set the reference date, starting proportion, and added relative fitness advantage based on such analyses to be used in the forecast. We generated those parameters at each day of the analysed trajectory, given the information of the VoC proportion on this day (see figure S1). The reference date for which the parameters were determined was defined as the first day where the proportion of the VoC exceeded 0.1%. We defined the VoCs based on the PANGO lineage classification for SARS-CoV2 variants where parent lineages are grouped with sub-lineages for which the same transmission properties are assumed [4] [5]. The WHO VoCs referenced in our analyses are Alpha (B.1.1.7 and Q lineages), Delta (B.1.617.2 and AY lineages), and Omicron (B.1.1.529 and BA lineages).

Immune evasion

In case the VoC has an advantage over the background variant through (partial) immune evasion, the number of individuals susceptible to the VoC ($S_V(t)$) would be greater than for the background variant ($S_B(t)$), giving it a higher $R_e(t)$ even if the $R_0(t)$ would remain the same.

We define immune evasion (η) as the proportion of individuals immune against infection with the background variant that are not immune against infection with the VoC. Thus, the number of individuals immune to infection with the VoC scales linearly with the number of individuals immune to infection with the background variant, if very few or no infections with the VoC have occured yet.

To correctly assess the added fitness advantage at the VoC reference point (t_ρ), we need to calculate the increase in the number of individuals susceptible to infection with the VoC relative to those susceptible to the background variant. For simplicity, we assume that the cumulative number of infections with the VoC is negligible relative to the cumulative number of infections with the background variants at t_ρ, that vaccination is aimed at the background variants, and

immunity against the VoC is only determined by cross-immunity $(1-\eta)$ from immunity against the background variants.

$$S_B(t_\rho = 0) = N(1 - G_P(t_\rho)) \left(1 - \sum_{i=0}^{t_\rho} \frac{I(i)}{N}\right)$$
$$S_V(t_\rho = 0) = N - (1-\eta)(N - S_B(t_\rho = 0))$$

Given that $R_{e,B}(t) = R_0(t)S_B(t)/N$ and $R_{e,V}(t) = R_0(t)(1+A)S_V(t)/N$, the relative added advantage by immune evasion, given the pre-existing immunity against the background variant, is

$$A_\eta = \frac{S_V(t_\rho)}{S_B(t_\rho)} - 1,$$

and the total added advantage is

$$\begin{aligned} A^* &= (1+A)(1+A_\eta) - 1, \\ &= (1+A)\frac{S_V(t_\rho)}{S_B(t_\rho)} - 1. \end{aligned}$$

As A^* is also the observed fitness advantage, the user will need to make an assumption about what part of this advantage is caused by immune evasion or by increased transmissibility on the reference date. Although the reference date (t_ρ) and the start of the simulation (T_S) are not necessarily the same, since the user can define t_ρ independently of T_S, from here on, we only consider the case where $T_S = t_\rho$, for clarity and brevity.

Incidence model and VoC

If a VoC is defined, we need to track the size of both the susceptible and the infected class of individuals for both the background variant and the VoC. This is first done retrospectively, for $t \leq T_S$, to determine the size of the classes at T_S.
With an estimate of the VoC proportion over time $(\rho_V(t \leq T_S))$, the past infections caused by the VoC can now be calculated as

$$I_V(t \leq T_S) = \lfloor \rho_V(t) I(t) \rceil,$$

where $\lfloor ... \rceil$ denotes rounding to the nearest integer. The number of infections with the background variants thus follows as $I_B(t) = I(t) - I_V(t)$. This also gives us the exact size of the susceptible class for both the background variant and the VoC

$$S_B(t) = N\left((1 - G_P(t))\left(1 - \sum_{i=0}^{t} \frac{I_B(i)}{N}\right)\right)\left(1 - (1-\eta)\sum_{i=0}^{t} \frac{I_V(i)}{N}\right),$$
$$S_V(t) = N\left((1 - (1-\eta)G_P(t))\left(1 - (1-\eta)\sum_{i=0}^{t} \frac{I_B(i)}{N}\right)\right)\left(1 - \sum_{i=0}^{t} \frac{I_V(i)}{N}\right).$$

Note that in the absence of new cases, the susceptible classes still need to be calculated forward in time before the incidence model is actually run, as forward vaccinations reduce their size.
The forward, prospective, model of the infection process, for $t > T_S$, is then performed for both the background variants and VoC in the same way as with a single variant, splitting $I(t)$, $P(t)$, $P_I(t)$, and $S(t)$ in a background variant $(*_B)$ and VoC $(*_V)$ class. The final epicurve then combines both variants again,

$$I(t) = I_B(t) + I_V(t).$$

Admission rate and VoC

In general, the assumption that the admission rate is stable over time reasonably holds unless the dynamics of the disease suddenly change. For now, we consider one such case: the occurrence of a variant with significant immune evasion, causing infections in individuals with immunity against the background variants through infection or vaccination (here called "reinfections", but it includes breakthrough infections to a large extent). In such a case, immunity in these individuals may protect against severe disease (to some degree). A sudden increase in the proportion of infections in these exposed individuals may lower the admission rate drastically.

We first need to calculate the number of reinfections. To do this, we track the proportion of individuals that have any immunity against any or either variant, assuming the second dose of the vaccine gives enough protection to reduce hospitalisation rates significantly,

$$U(t) = 1 - \left(1 - \sum_{i=0}^{T_S} \frac{I(i)}{N}\right)\left(1 - \sum_{i=0}^{T_S} \frac{V_2(i)}{N}\right),$$

$$U_B(t) = 1 - \left(1 - \sum_{i=0}^{T_S} \frac{I_B(i)}{N}\right)\left(1 - \sum_{i=0}^{T_S} \frac{V_2(i)}{N}\right),$$

$$U_V(t) = \sum_{i=0}^{T_S} \frac{I_V(i)}{N}.$$

The number of reinfections is then determined by comparing the susceptible class (S) with the previous immunity class (U). The sizes of these classes differ because of non-complete protection from vaccination and cross-immunity between strains.

$$X_B(t) = \frac{S_B(t) - (1 - U_B(t))(1 - U_V(t))N}{S_B(t)} I_B(t),$$

$$X_V(t) = \frac{S_V(t) - (1 - U_B(t))(1 - U_V(t))N}{S_V(t)} I_V(t).$$

The proportion of reinfections among all infections is thus

$$X^*(t) = \frac{X_B(t) + X_V(t)}{I(t)}$$

The admission rate can then be adjusted to consider the effect of reinfections. This is done by adjusting future admission rates relative to the observed admission rate at the start of the simulation. First, we calculate the risk of admission for immunologically naive individuals (the "Primary hospitalisation risk"), π, at the start of the simulation

$$\pi = \frac{\alpha(T_S)}{(1 - X^*(T_S)) + (\gamma X^*(T_S))},$$

given the relative admission risk for secondary infections γ. The admission rate over time then develops with $X^*(t)$

$$\alpha^*(t) = X^*(t)\pi\gamma + (1 - X^*(t))\pi$$

Development of Variants of Concern over time

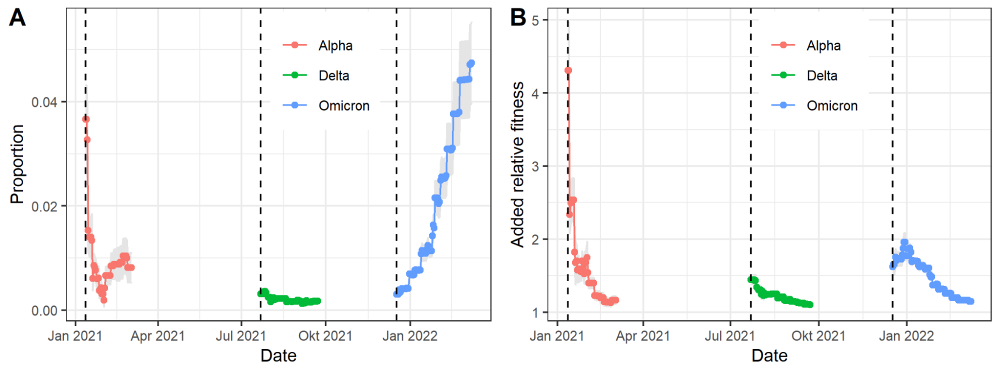

Figure S1: **Development of VoCs.** Estimated proportion (A) and its corresponding added relative fitness advantage (B) of the Alpha, Delta, and Omicron variants in regard to the previously prevailing variant in Germany for a reference date. The reference dates for the VoCs are marked as dashed vertical lines. The grey area shows the 95% interval range.

S2 ETS model

To produce a dynamic forecast of $R_0(t)$, we used an ETS model which underlies an exponential smoothing method [6]. In general, exponential smoothing methods generate point forecasts of time series using weighted averages.
For an observed time series $y_1, y_2, ..., y_t$ the point forecast for h periods ahead is y_{t+h}. When the forecast is based on all data up to time t, it is denoted as $y^*_{t+h|t}$. For a method with an additive trend, the point forecast results from the level of the series l and the growth b at time t:

$$y^*_{t+h|t} = l_t + hb_t$$

which gives the forecast equation, while l_t and b_t are based on the level and trend (slope) at $t-1$ and are given by the smoothing equations:

$$l_t = \alpha y_t + (1-\alpha)(l_{t-1} + b_{t-1}),$$
$$b_t = \beta^*(l_t - l_{t-1}) + (1-\beta^*)b_{t-1}.$$

This approach is also known as Holt's linear method. Values for the initial states l_0 and b_0 as well as the smoothing parameters α and β^* are estimated from the observed data, with $0 < \alpha, \beta^* < 1$. Increasing α gives more weight to the more recent observations and less weight to observations that lie longer ago. In our case, α and β^* were set to 0.25 and 0.15, respectively.
While the exponential smoothing methods generate point forecasts, the underlying statistical models, in addition, generate prediction intervals. To generate such an ETS forecast model, we need to specify the probability distribution for the residual at time t, e_t. For additive errors, we assume the residuals are normally and independently distributed with mean 0 and variance σ^2, notated as $e_t = \varepsilon_t \sim \mathrm{NID}(0, \sigma^2)$. Expressing the error as $\varepsilon_t = y_t - l_{t-1} - b_{t-1} \sim \mathrm{NID}(0, \sigma^2)$ we can substitute ε_t into Holt's linear method

$$y_t = l_{t-1} + b_{t-1} + \varepsilon_t,$$

$$l_t = l_{t-1} + b_{t-1} + \alpha\varepsilon_t,$$
$$b_t = b_{t-1} + \beta^{**}\varepsilon_t.$$

with $\beta^{**} = \alpha\beta^*$. These expressions are referred to as the state-space models as they include an equation for the observation and equations for the unobserved states (level, trend). Each observation y_t in the ETS model therefore includes a component for the level (or smoothed value), an error component (E), a trend component (T), and seasonality (S). In our case, we use the ETS(A,A,N) model, which has additive errors, additive trend and no seasonality.

We use a $\log(R_0(t))$ time series of a 100 days, i.e. $\log(R_0(T_S - 99...T_S))$, as input for the ETS model. The logarithm serves to forecast the relative changes in R_0 and avoids negative R_0 forecasts. Per model iteration, we pick a random quantile for the prediction interval and use this as the trajectory for $R_0(t > T_S)$.

S3 Benchmark of dashboard performance

Between 23-12-2021 and 07-04-2022, the online dashboard was visited 2352 times (counted as the number of unique sessions) by 575 users (counted as the number of unique machines by cookie UUID (Universally Unique Identifier)). On average, sessions lasted 22 minutes, but the distribution of session durations is heavily right-skewed, ranging between 0 seconds (i.e., immediately closed by the user) and 12 hours, and a median of 1.6 minutes (IQR 0.3 - 5.9 minutes). There were active sessions during 471 hours of the 2523 hours.

For 219 hours, the dashboard served multiple concurrent users, specifically more than three users for 44.5 hours, to a maximum of six users on 23-12-2021 and 19-01-2022.

Users primarily selected hospital catchments consisting of a single district (n=2507), followed by 44 districts (N=1426), coinciding with the number of districts in the state of Baden-Wrttemberg, and 412 districts (N=807), indicating all districts in Germany. The incidence model was called 6875 times and took 2.7s on average (median 1.4s IQR 0.8-2.5s), with a strong dependency on the total number of run-days (number of runs × length of the simulation, see figure S2). The care path model was called 5735 times and took 4.2s on average (median 1.9s IQR 0.9-4.1s).

As with the incidence model, the run time of the care path model depended strongly on the number of run-days. Additionally, it depends on the total number of beds occupied at the start of the simulation. On 157 occasions, users uploaded a file specifying the number of beds occupied in a specific hospital. This uploading was done by 24 unique users, of which two were responsible for the vast majority of uploading instances (74 and 33 times). The other forecasts were thus based on the number of beds occupied in each of the districts as automatically loaded from the central database.

This work shows that hospital-specific on-request production of bed demand forecasts during pandemics is possible. The developed on-request forecasting tool offers users maximum flexibility by allowing the definition of hospital-specific assumptions and/or starting conditions, while at the same time basing all forecasts on the same model. The combination of user-defined inputs within an existing model framework allows public health experts to adapt advanced forecasting methods to their local setting without having to build such models from scratch. Such an approach has the potential to make mathematical modelling of infectious diseases available to a much larger group of stakeholders, each with their specific set of questions to be answered.

Although our dashboard had a relatively modest number of users during the study duration, we are confident that it can cope with many more users, given the low proportion of time concurrent users were present. Nonetheless, there is certainly a limit to the traffic capacity of the dashboard, and particularly peak demand may be challenging. For this reason, we avoided publicly promoting our dashboard, relying instead on our network of hospitals and other healthcare

organisations to circulate it. However, the dashboard was openly and freely available to any user over the entire period. Furthermore, the code is freely available and can be installed and run locally if hospitals need their own instance of the dashboard.

In conclusion, on-request forecasts are a fundamentally different method of providing infectious disease forecasts compared to the more common static reports. We showed how our dashboard implementation solves the specific set of challenges related to open-ended dynamic forecasting platforms, in particular computational requirements. The dashboard was successfully used by local healthcare providers, hospitals, and healthcare policymakers to evaluate incidence and hospital bed occupancy in Germany during the 2020-2022 COVID-19 pandemic. We argue that these on-request forecasts are much more helpful in informing stakeholders at a local level where health management decisions, such as cancelling elective surgeries, directly affect the bed capacity. This way, the pandemic or epidemic response can be driven in near real-time on the level where it matters most.

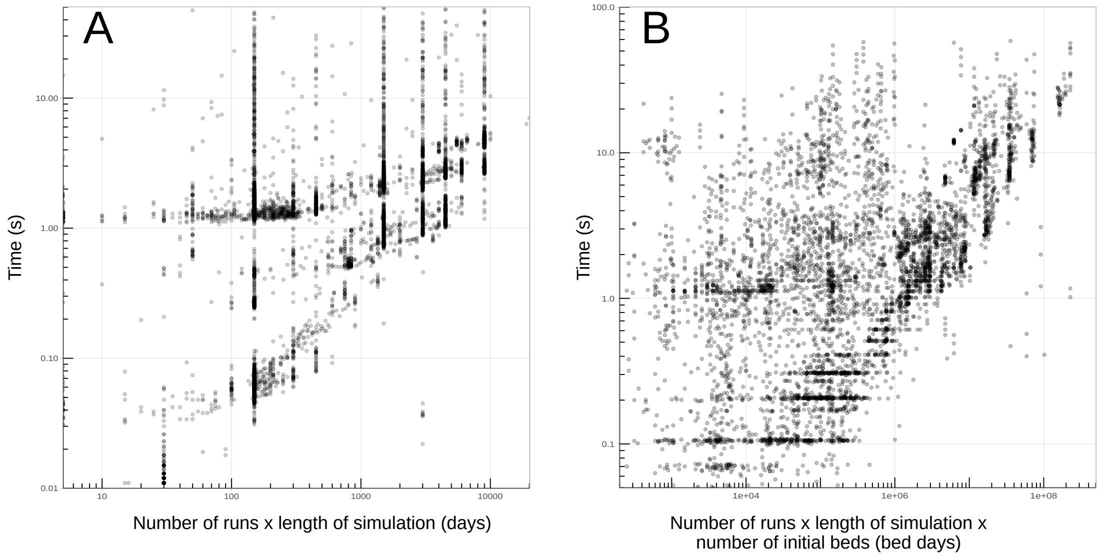

Figure S2: **The run-time of (A) the incidence model and (B) the care path model, as a function of the total number of run-days and bed-run-days, respectively.**

S4 Forecast of $R_e(t)$ for Freiburg catchments

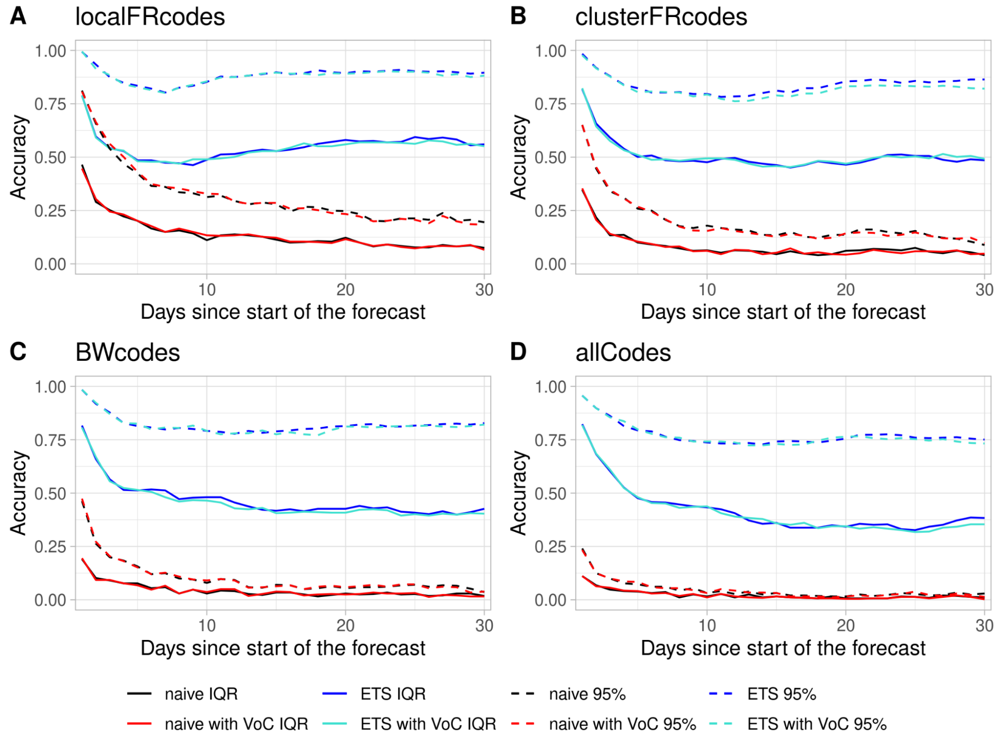

Figure S3: **Accuracy of the $R_e(t)$ forecast.** Accuracy is shown for (A) local Freiburg catchment, (B) the Freiburg cluster, (C) Baden-Wrttemberg, (D) and whole Germany over 30 days. Solid lines represent accuracy based on the interquartile range (IQR) while dashed lines represent accuracy based on the 95% range. The $R_e(t)$-value was predicted using the naive forecast (black lines), naive forecast including VoC (red lines), exponential smoothing (ETS) (dark blue lines), and exponential smoothing including VoC (light blue lines).

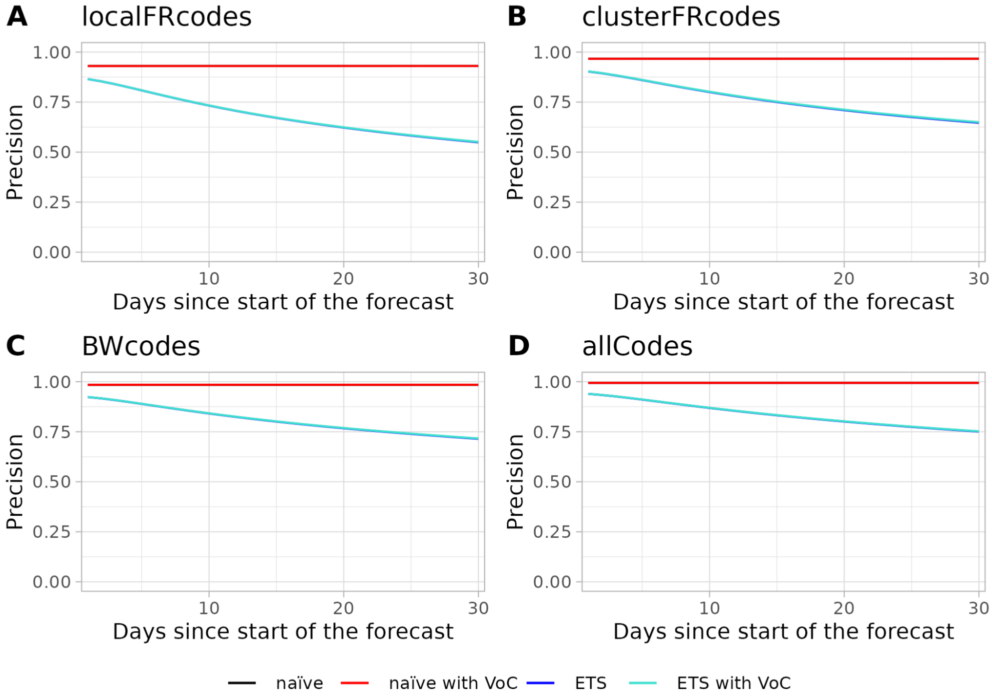

Figure S4: **Precision of the $R_e(t)$ forecast. Precision is shown for (A) local Freiburg catchment, (B) the Freiburg cluster, (C) Baden-Wrttemberg, (D) and whole Germany over 30 days.** The $R_e(t)$-value was predicted using the naive forecast (black lines), naive forecast including VoC (red lines), exponential smoothing (ETS) (dark blue lines), and exponential smoothing including VoC (turquoise lines).

S5 Evaluation of bed demand forecasts

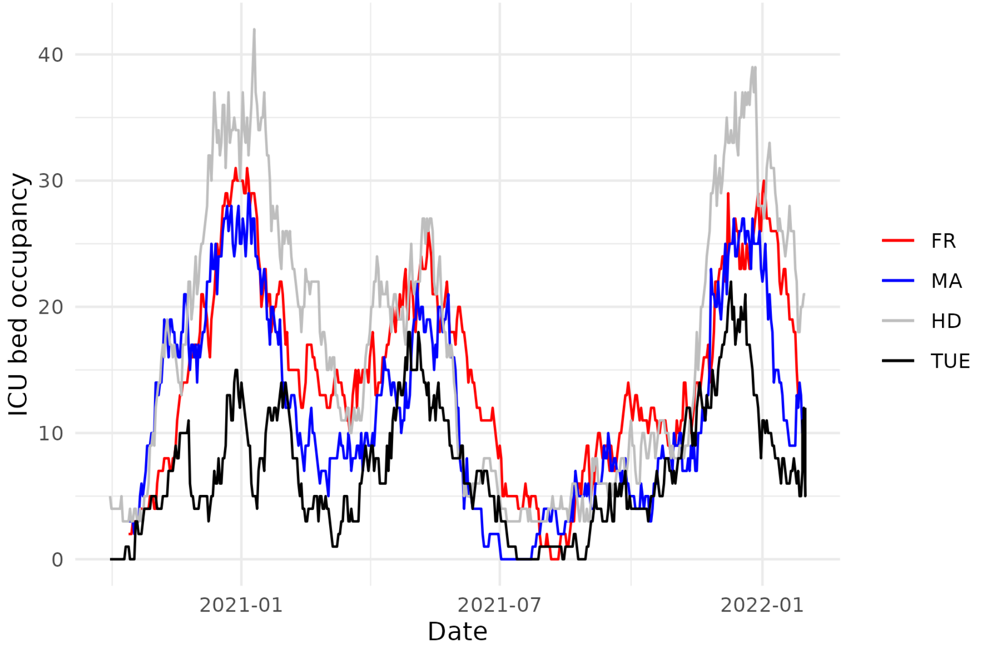

Figure S5: **Bed occupancy of ICU units.** Bed occupancy is shown for university hospitals in Freiburg (FR), Mannheim (MA), Heidelberg (HD), and Tbingen (TUE) over time.

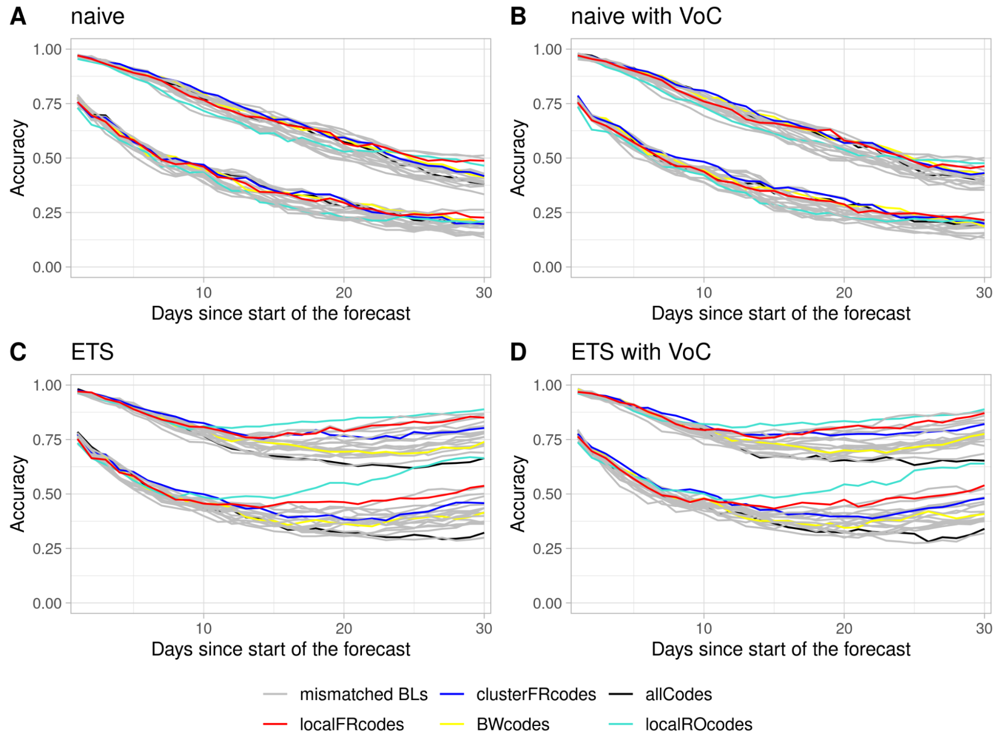

Figure S6: **Accuracy of the ICU bed forecast for 30 days based on the bed occupancy of the University Hospital in Freiburg for different catchment areas.** Forecasts are based on different methods for predicting the $R_e(t)$-value: naive (A), naive including Variant of Concern (VoC) (B), exponential smoothing (ETS) (C), and exponential smoothing including VoC. The lower bundle of lines represents accuracy based on the interquartile range (IQR) while the upper bundle represents accuracy based on the 95% confidence range.

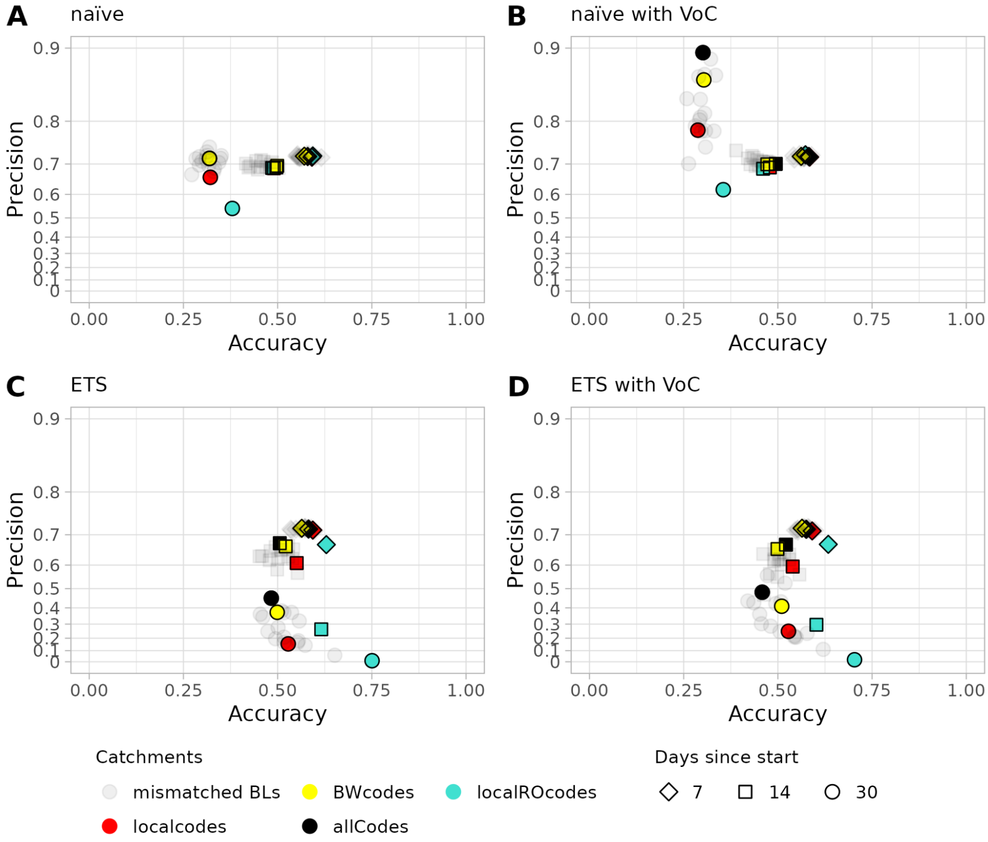

Figure S7: **ICU bed forecast based on the bed occupancy of the University Hospital in Mannheim.** Precision versus accuracy of ICU bed forecasts based on the bed occupancy of the University Hospital in Freiburg is shown. Forecasts are based on different methods for the predicted $R_e(t)$-value: naive (A), naive including Variant of Concern (VoC) (B), exponential smoothing (ETS) (C), and exponential smoothing including VoC. Colours represent different catchment areas (red: Freiburg local, blue: Freiburg cluster, yellow: Baden-Wrttemberg (BW), turquoise: Rostock, black: whole Germany, grey: all states separately except BW). Diamond shapes represent the 7th day, square shapes the 14th day, and circles the 30th day of the forecasts.

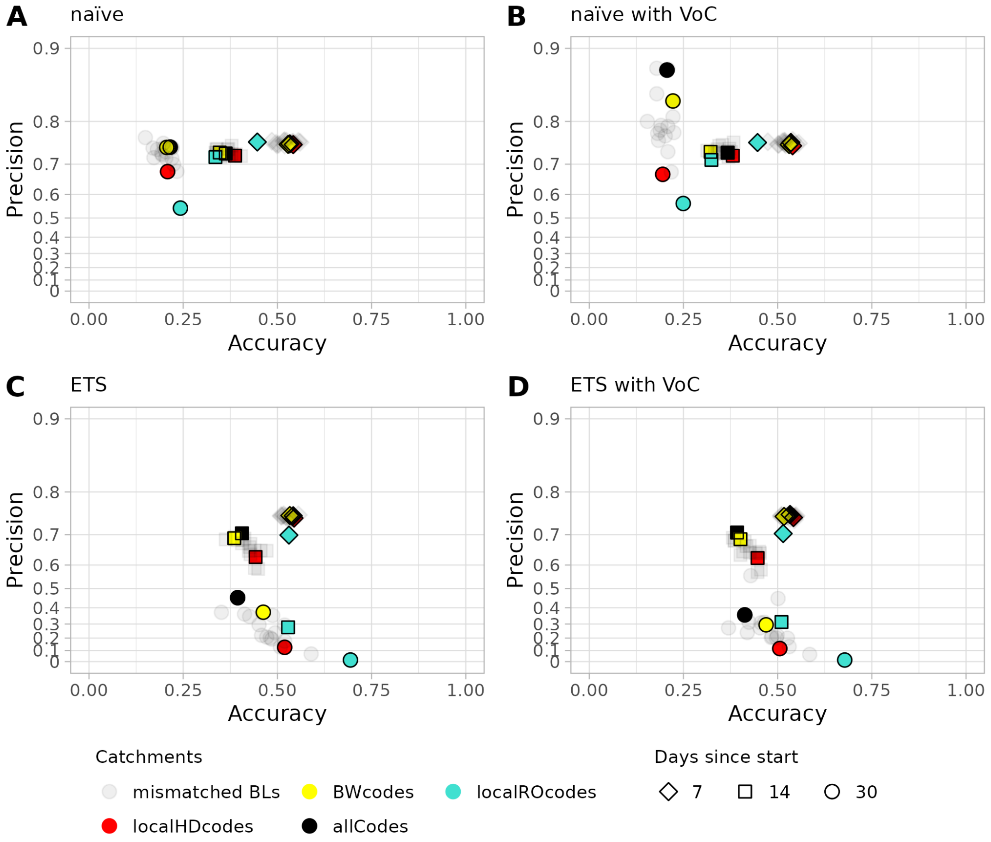

Figure S8: **ICU bed forecast based on the bed occupancy of the University Hospital in Heidelberg.** Precision versus accuracy of ICU bed forecasts based on the bed occupancy of the University Hospital in Freiburg is shown. Forecasts are based on different methods for the predicted $R_e(t)$-value: naive (A), naive including Variant of Concern (VoC) (B), exponential smoothing (ETS) (C), and exponential smoothing including VoC. Colours represent different catchment areas (red: Freiburg local, blue: Freiburg cluster, yellow: Baden-Wrttemberg (BW), turquoise: Rostock, black: whole Germany, grey: all states separately except BW). Diamond shapes represent the 7th day, square shapes the 14th day, and circles the 30th day of the forecasts.

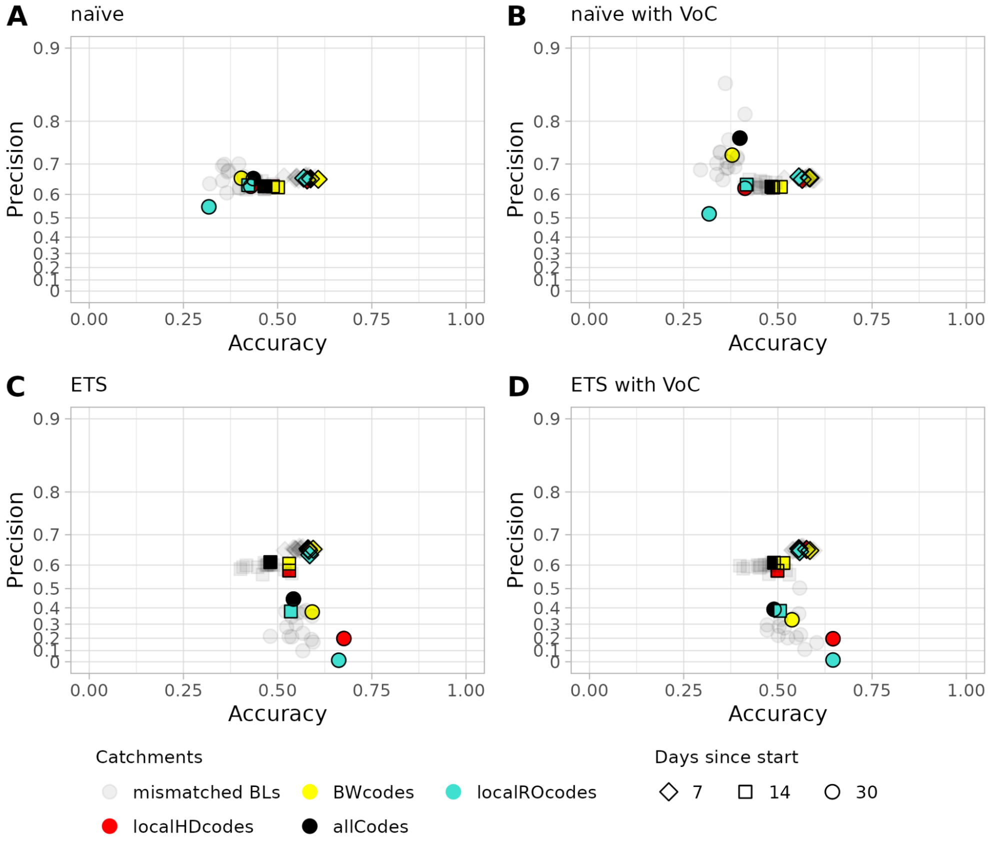

Figure S9: **ICU bed forecast based on the bed occupancy of the University Hospital in Tbingen.** Precision versus accuracy of ICU bed forecasts based on the bed occupancy of the University Hospital in Freiburg is shown. Forecasts are based on different methods for the predicted $R_e(t)$-value: naive (A), naive including Variant of Concern (VoC) (B), exponential smoothing (ETS) (C), and exponential smoothing including VoC. Colours represent different catchment areas (red: Freiburg local, blue: Freiburg cluster, yellow: Baden-Wrttemberg (BW), turquoise: Rostock, black: whole Germany, grey: all states separately except BW). Diamond shapes represent the 7th day, square shapes the 14th day, and circles the 30th day of the forecasts.

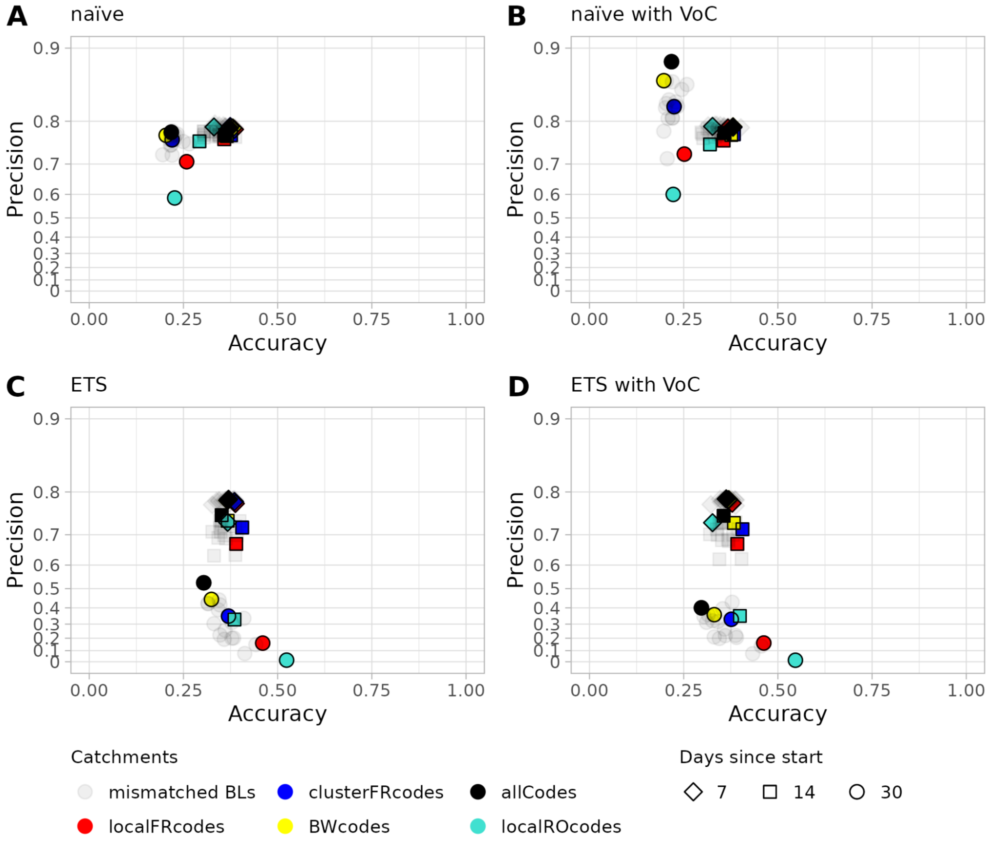

Figure S10: **General Ward bed forecast based on the bed occupancy of the University Hospital in Freiburg.** Precision versus accuracy of General Ward bed forecasts based on the bed occupancy of the University Hospital in Freiburg is shown. Forecasts are based on different methods for the predicted $R_e(t)$-value: naive (A), naive including Variant of Concern (VoC) (B), exponential smoothing (ETS) (C), and exponential smoothing including VoC. Colours represent different catchment areas (red: Freiburg local, blue: Freiburg cluster, yellow: Baden-Wrttemberg (BW), turquoise: Rostock, black: whole Germany, grey: all states separately except BW). Diamond shapes represent the 7th day, square shapes the 14th day, and circles the 30th day of the forecasts.

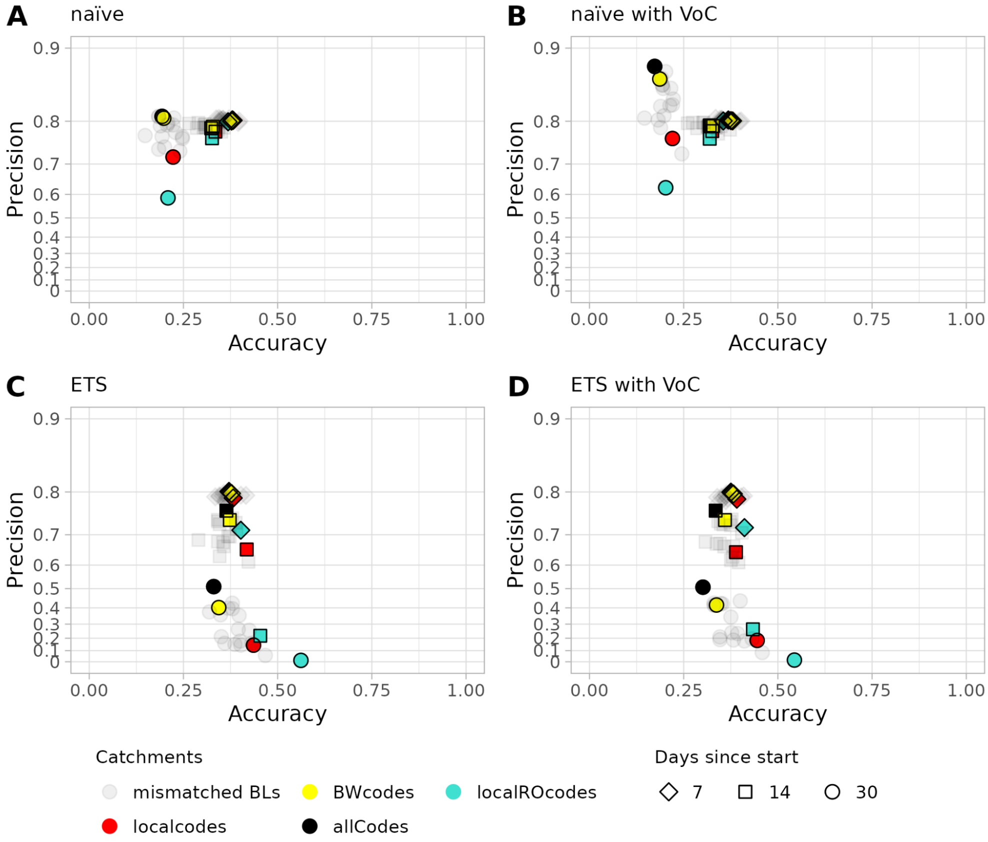

Figure S11: **General Ward bed forecast based on the bed occupancy of the University Hospital in Mannheim.** Precision versus accuracy of General Ward bed forecasts based on the bed occupancy of the University Hospital in Freiburg is shown. Forecasts are based on different methods for the predicted $R_e(t)$-value: naive (A), naive including Variant of Concern (VoC) (B), exponential smoothing (ETS) (C), and exponential smoothing including VoC. Colours represent different catchment areas (red: Freiburg local, blue: Freiburg cluster, yellow: Baden-Wrttemberg (BW), turquoise: Rostock, black: whole Germany, grey: all states separately except BW). Diamond shapes represent the 7th day, square shapes the 14th day, and circles the 30th day of the forecasts.

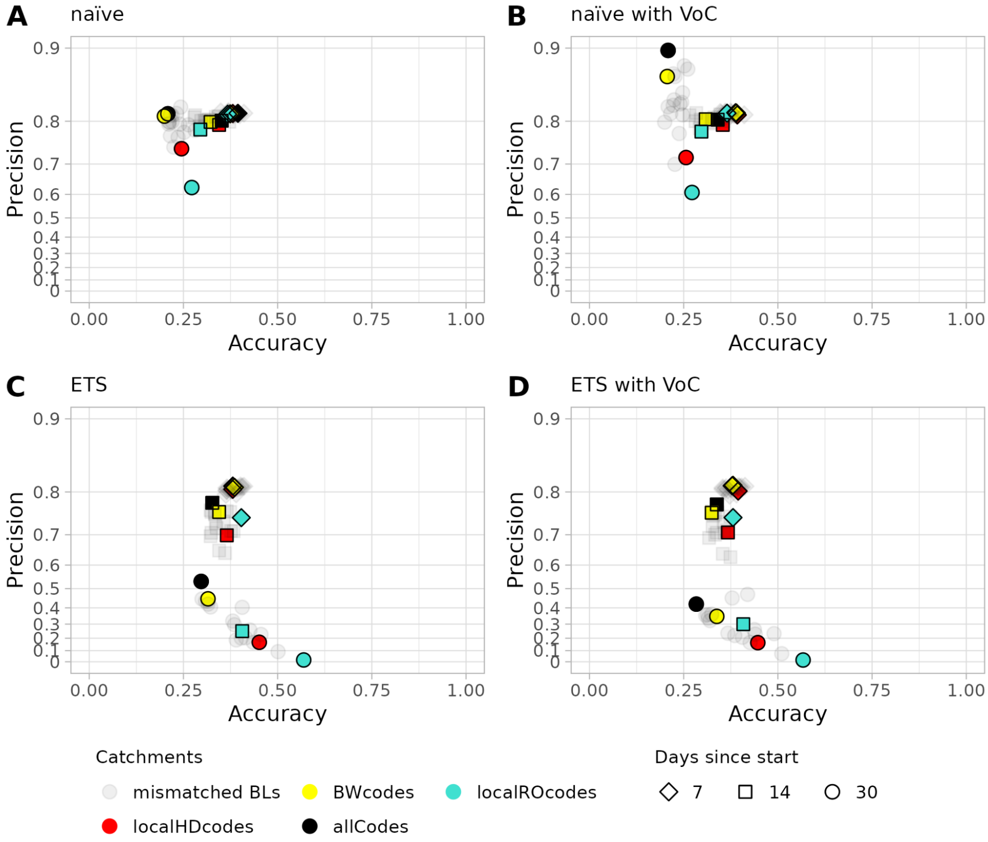

Figure S12: **General Ward bed forecast based on the bed occupancy of the University Hospital in Heidelberg.** Precision versus accuracy of General Ward bed forecasts based on the bed occupancy of the University Hospital in Freiburg is shown. Forecasts are based on different methods for the predicted $R_e(t)$-value: naive (A), naive including Variant of Concern (VoC) (B), exponential smoothing (ETS) (C), and exponential smoothing including VoC. Colours represent different catchment areas (red: Freiburg local, blue: Freiburg cluster, yellow: Baden-Wrttemberg (BW), turquoise: Rostock, black: whole Germany, grey: all states separately except BW). Diamond shapes represent the 7th day, square shapes the 14th day, and circles the 30th day of the forecasts.

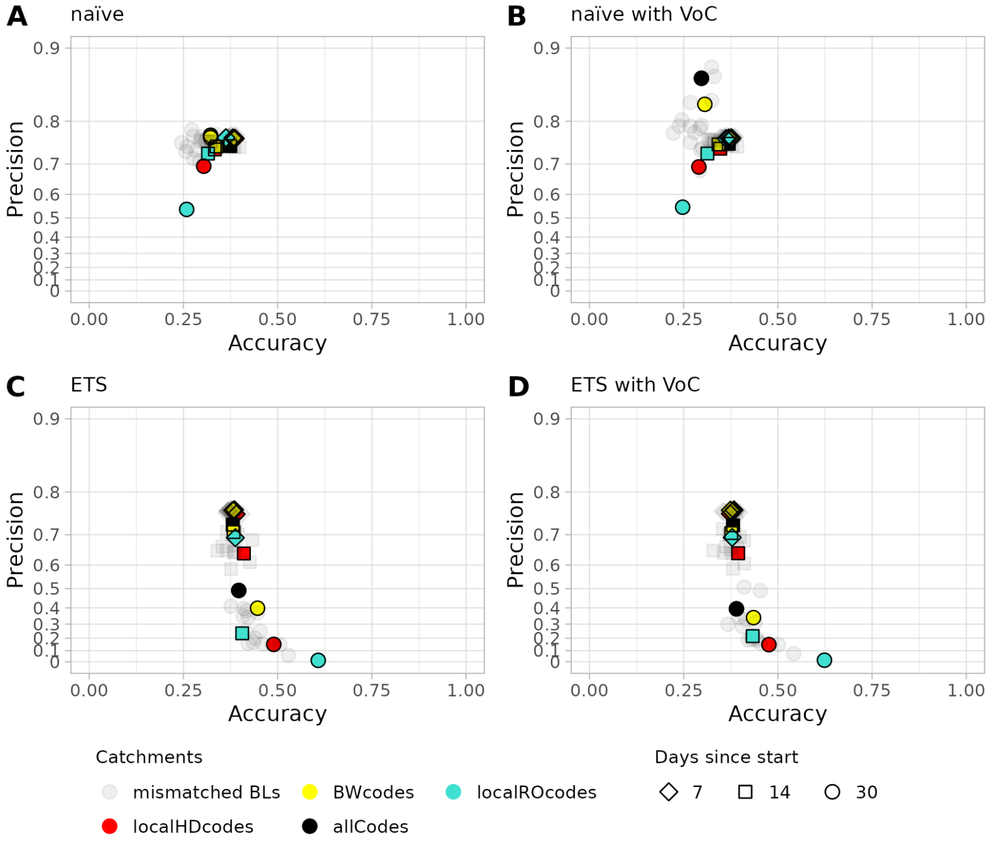

Figure S13: **General Ward bed forecast based on the bed occupancy of the University Hospital in Tbingen.** Precision versus accuracy of General Ward bed forecasts based on the bed occupancy of the University Hospital in Freiburg is shown. Forecasts are based on different methods for the predicted $R_e(t)$-value: naive (A), naive including Variant of Concern (VoC) (B), exponential smoothing (ETS) (C), and exponential smoothing including VoC. Colours represent different catchment areas (red: Freiburg local, blue: Freiburg cluster, yellow: Baden-Wrttemberg (BW), turquoise: Rostock, black: whole Germany, grey: all states separately except BW). Diamond shapes represent the 7th day, square shapes the 14th day, and circles the 30th day of the forecasts.

S6 MASE and bias calculation

A metric to evaluate the performance of the forecasts is the mean absolute scaled error (MASE)[16]:

$$MASE(d) = \frac{\frac{1}{J}\sum_j |e_{ij}|}{\frac{1}{J}\sum_j |Y_{d,i} - Y_{d,j=0}|} \quad (1)$$

Here, the numerator e_j is the forecast error defined as the actual value Y_j minus the forecasted value F_j : $e_j = Y_j - F_j$ with J being the number of forecasted values. Here, the forecast was established using ETS. The denominator is the absolute mean error based on the one-step naive

forecast method, which equals F_j to the previous observation Y_{j-1}. We applied MASE on the median of 100 parallel model runs from each forecast to analyse the time dependency of the $R_e(t)$ forecast accuracy.
Under- and overestimation of the bed forecasts was estimated using the bias:

$$bias(d) = \frac{1}{J}\sum_j F_{d,j} - Y_{d,j} \tag{2}$$

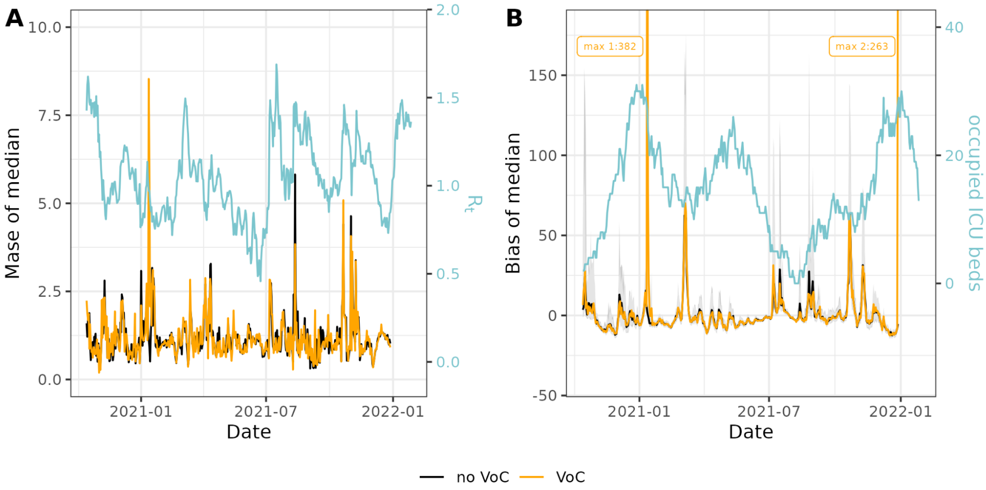

Figure S14: **Mean absolute scaled error (MASE) of the median of $R_e(t)$ forecasts and the bias of the median of the occupied ICU beds.** Results for the university hospital of Freiburg using the local FR catchment with and without VoC are shown. The medians of 100 model runs for each forecast are given. (a) The median of MASE is contrasted with the observed $R_e(t)$ values (right axis, light blue). (b) Values of the two highest peaks are indicated in the orange boxes.

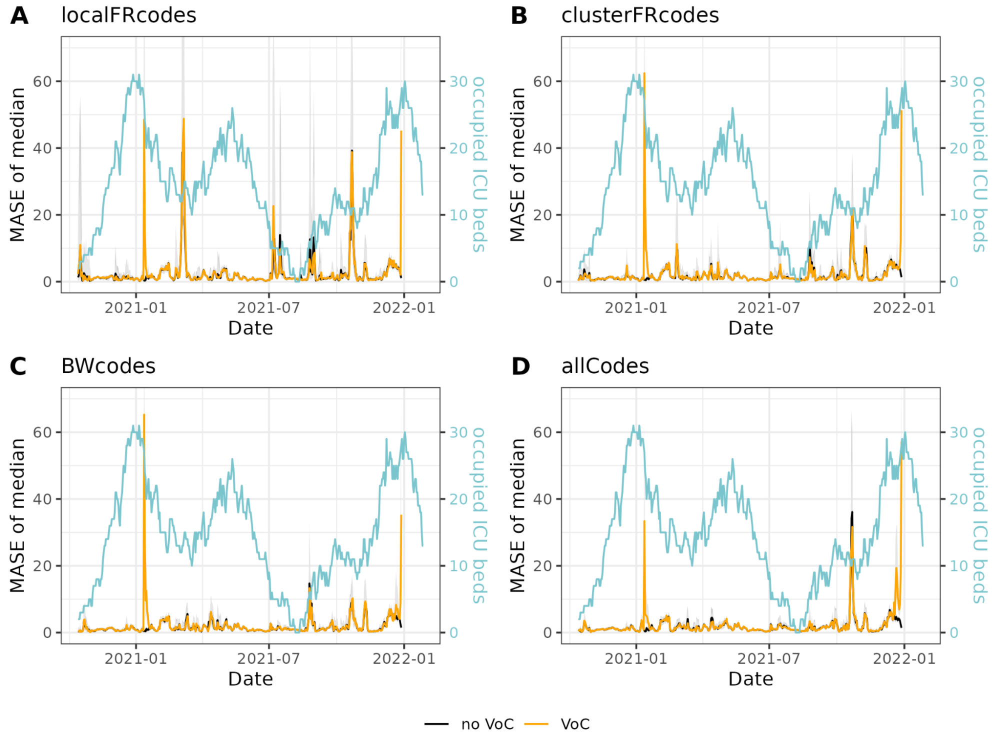

Figure S15: **Median of the mean absolute scaled error (MASE) of forecasts of ICU bed occupancy for university hospital of Freiburg with and without Variant of Concern (VoC).** Results are shown for Freiburg (A), Baden-Wrttemberg (B), all states combined (C), and whole of Germany (D). The median of MASE is contrasted with the observed ICU bed occupancy in the university hospital of Freiburg (right axis, light blue).

S7 Forecast evaluation for Rostock

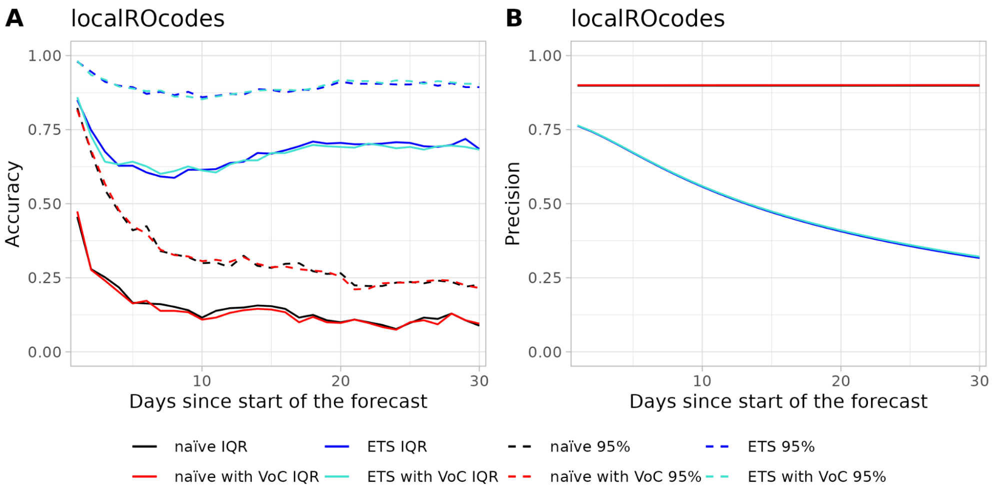

Figure S16: **Evaluation of the $R_e(t)$ forecast for Rostock.** Shown are accuracy (A) and precision (B) of the $R_e(t)$ forecast for Rostock. The $R_e(t)$-value was predicted using the naive forecast (black line), naive forecast including VoC (red line), exponential smoothing (blue line), and exponential smoothing including VoC (turquoise line).

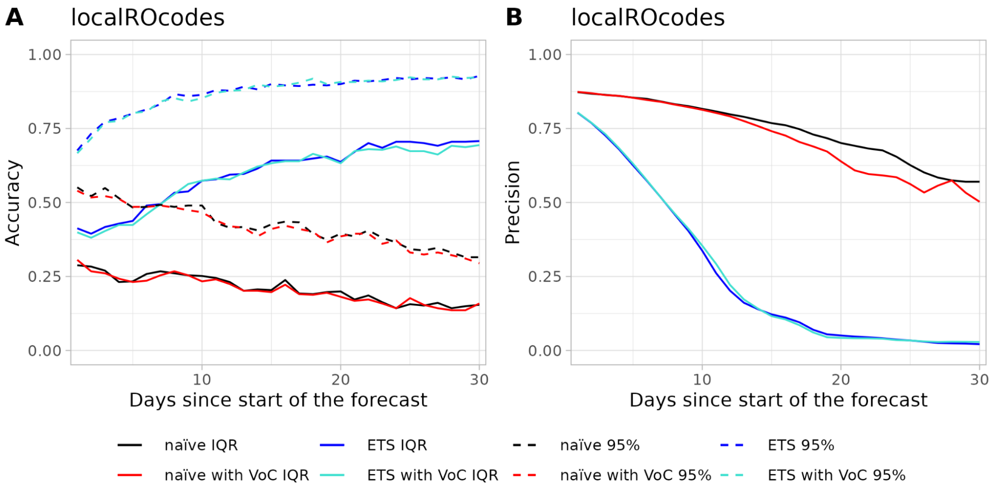

Figure S17: **Evaluation of the incidence forecast for Rostock.** Shown are accuracy (A) and precision (B) of the incidence forecast for Rostock. The $R_e(t)$-value was predicted using the naive forecast (black line), naive forecast including VoC (red line), exponential smoothing (blue line), and exponential smoothing including VoC (turquoise line).

References

[1] Abdool Karim, S.S., de Oliveira, T.: New SARS-CoV-2 Variants Clinical, Public Health, and Vaccine Implications. New England Journal of Medicine **384**(19), 1866–1868 (2021). doi:10.1056/NEJMc2100362. Publisher: Massachusetts Medical Society _eprint: https://doi.org/10.1056/NEJMc2100362. Accessed 2022-05-05

[2] Althaus, C.L., Baggio, S., Reichmuth, M.L., Hodcroft, E.B., Riou, J., Neher, R.A., Jacquerioz, F., Spechbach, H., Salamun, J., Vetter, P., Williamson, C., Hsiao, N.-y., Preiser, W., Davies, M.-A., Lessells, R.J., de Olivera, T., Kaiser, L., Eckerle, I.: A tale of two variants: Spread of SARS-CoV-2 variants Alpha in Geneva, Switzerland, and Beta in South Africa. preprint, Epidemiology (June 2021). doi:10.1101/2021.06.10.21258468. http://medrxiv.org/lookup/doi/10.1101/2021.06.10.21258468 Accessed 2022-03-11

[3] Wallinga, J., Lipsitch, M.: How generation intervals shape the relationship between growth rates and reproductive numbers. Proceedings of the Royal Society B Biological Sciences **274**(1609), 599–604 (2007). doi:10.1098/rspb.2006.3754. Publisher: Department of Infectious Diseases Epidemiology, National Institute of Public Health and the Environment, PO Box 1, 3720 BA Bilthoven, The Netherlands. jacco.wallinga@rivm.nl. Accessed 2011-06-27

[4] Rambaut, A., Holmes, E.C., OToole, Á., Hill, V., McCrone, J.T., Ruis, C., du Plessis, L., Pybus, O.G.: A dynamic nomenclature proposal for sars-cov-2 lineages to assist genomic epidemiology. Nature microbiology **5**(11), 1403–1407 (2020)

[5] Rambaut, A., Holmes, E.C., OToole, Á., Hill, V., McCrone, J.T., Ruis, C., du Plessis, L., Pybus, O.G.: Addendum: A dynamic nomenclature proposal for sars-cov-2 lineages to assist genomic epidemiology. Nature microbiology **6**(3), 415–415 (2021)

[6] Hyndman, Rob J., Athanasopoulos, George: Forecasting: Principles and Practice (3rd ed), (2021). https://otexts.com/fpp3/ Accessed 2022-05-05
